# Phage-resistance alters Lipid A reactogenicity: a new strategy for LPS-based conjugate vaccines against *Salmonella* Rissen

**DOI:** 10.3389/fimmu.2024.1450600

**Published:** 2024-12-11

**Authors:** Paola Cuomo, Chiara Medaglia, Angela Casillo, Antonio Gentile, Carmine Fruggiero, Maria Michela Corsaro, Rosanna Capparelli

**Affiliations:** ^1^ Department of Agricultural Sciences, University of Naples Federico II, Portici, Italy; ^2^ Task Force on Microbiome Studies, University of Naples Federico II, Naples, Italy; ^3^ Functional Genomics Research Center, Fondazione Human Technopole, Milan, Italy; ^4^ Department of Chemical Sciences, University of Naples Federico II, Naples, Italy

**Keywords:** bacteriophages, host immune response, lipid A, salmonella infection, vaccine

## Abstract

*Salmonella enterica* serovar Rissen (*S.* Rissen) is an emerging causative agent of foodborne diseases. The current emergence of antibiotic resistance makes necessary alternative therapeutic strategies. In this study, we investigated the potential of a phage-resistant strain of *S.* Rissen (R^R^) as a tool for developing an effective lipopolysaccharide (LPS)-based vaccine. The LPS O-antigen is known to play critical roles in protective immunity against *Salmonella*. However, the high toxicity of the LPS lipid A moiety limits its use in vaccines. Here, we demonstrated that the acquisition of bacteriophage resistance by *S.* Rissen leads to structural modifications in the LPS structure. Using NMR and mass spectrometry, we characterized the LPS from phage-resistant strains as a smooth variant bearing under-acylated Lipid A portions (penta- and tetra-acylated forms). We then combined RT-qPCR and NMR-based metabolomics to explore the effects of phage resistance and LPS modification on bacterial fitness and virulence. Finally, we conducted *in vivo* studies to determine whether lysogeny-induced remodeling of LPS affects the host immune response. Results revealed that the under-acylated variant of LPS from *R^R^* attenuates the inflammatory response in BALB/c mice, while eliciting a specific antibody response that protects against *S.* Rissen (R^W^) infection. In conclusion, our findings suggest that phage resistance, through lipid A modification, may offer a novel strategy for reducing LPS toxicity, highlighting its potential as a promising biological approach for developing LPS-based vaccines against *Salmonella* infections.

## Introduction

Salmonellosis is a major enteric infection causing a high rate of mortality worldwide every year (1, 2). The limited treatment options due to antimicrobial resistance and the absence of an effective prophylactic therapy, have contributed to increase the annual morbidity and mortality, positioning salmonellosis as a serious public health concern (3). In the last few years, *S.* Rissen has emerged as a notable serovar causing salmonellosis in various regions of the world (4), making the development of a successful vaccine a pressing challenge.

To date, only three categories of vaccine are licensed for protection against *Salmonella* infection, and specifically *S*. typhi (5). These are the live attenuated vaccine Ty21a, a subunit vaccine based on the Vi capsular polysaccharide (Vi CPS) and the Vi typhoid conjugate vaccine (TCV) (6). Despite their different approaches, both Ty21a and unconjugated Vi CPS vaccines have severe limitations. Neither Ty21a nor unconjugated Vi CPS provide protection for young children, and they offer limited efficacy in adults (7). Additionally, Ty21a contains a mixture of antigens which makes difficult to determine which specific antigen confers protection or increases the risk of adverse effects (8). These difficulties have been successfully overcome by TCV, which promises long-lasting immunity and protection in infants while maintaining a favorable safety profile (9). However, TCV also has a limitation as it confers protection only against a selected subset of *Salmonella* species expressing the Vi capsular polysaccharide. Consequently, the growing interest in developing *Salmonella* vaccines has led to the exploration of alternative vaccine strategies. Most of these strategies, including the Generalized Modules for Membrane Antigens (GMMA), focus on targeting the O-antigen of the lipopolysaccharide (LPS) (10, 11).

LPS, also known as endotoxin, is a glycolipid located in the outer membrane of Gram-negative bacteria (12). It consists of a conserved Lipid A, a core oligosaccharide and a variable O-polysaccharide (OPS) (13). For many years, the polysaccharide component of LPS was thought to lack antigenicity. This assumption was definitively disproved by André Boivin, who demonstrated that isolated polysaccharide moieties can act as haptens, retaining antigenic properties without stimulating antibody production (14). Boivin also showed that smooth bacteria, which possess a “complete antigen”, composed of a complete OPS and Lipid A, stimulate robust immune responses by activating the Toll-like receptor 4 (TLR4) and producing polysaccharide-specific antibodies that neutralize pathogenic bacteria (15). As a result, smooth LPS (sLPS) is a virulence factor and a valid target for vaccine development, compared to rough LPS (rLPS) - which lack OPS or contain only a single subunit (16).

sLPS has been proven to be particularly effective in preventing infections caused by various *Enterobacteriaceae*, such as *Klebsiella pneumoniae*, *Shigella flexneri* and *Salmonella* (17–19). However, due to the diversity of O-antigens among Enterobacteria, sLPS is expected to induce an immune response with restricted specificity. Interestingly, the high similarity of surface molecules among *Salmonella* strains makes sLPS an attractive candidate for broader-spectrum vaccines capable of cross-reactive immunity and protection against closely related *Salmonella* serotypes sharing analogous O-antigen profiles (20). Despite this, the high toxicity of sLPS, due to Lipid A component, makes it challenging to develop safe vaccines.

To address this concern and develop safe and immunogenic vaccines, several approaches have been attempted. Among these, genetic manipulation for silencing genes encoding acyl transferases, such as *msbB*, *htrB* and *pagP*, has been the most successful (21, 22). This results in mutant strains that - compared to the parent strain - synthetize LPS molecules with different lengths or numbers of fatty acyl chains in Lipid A, leading to attenuation of the LPS signaling pathway and reduced LPS toxicity (23, 24).

In a previous study, we demonstrated that LPS from the bacteriophage-resistant strain of *S.* Rissen (R^R^) induces a reduced inflammatory response *in vitro* compared to the LPS produced by the bacteriophage-sensitive strain of *S*. Rissen (R^W^) (25, 26), and attributed this result to Lipid A modification.

The goal of the present study was to validate our hypothesis, proposing bacteriophage-resistance as an alternative strategy to traditional chemical, enzymatic, or genetic manipulations, to obtain under-acylated variants of Lipid A, useful for producing LPS-based vaccines.

Lysogenic phages play an influential role in shaping various aspects of bacterial lifestyle, significantly contributing to both micro- and macro-diversity observed within bacterial species (27). Of note, prophage Φ1 has been found to introduce variability between R^R^ and R^W^ strains. Unlike R^W^, the R^R^ strain demonstrated a propensity to spontaneously lose the phage Φ1 without generating phage particle. Yet, the acquisition of phage-resistance has been found to supply the R^R^ strain with additional virulence factors (26). To realize the above transformations, the phage Φ1 provides the host new genetic material which confers several advantages (25).

Morons are prophage genes encoding proteins that enhance bacterial fitness through different mechanisms (28). They can increase bacterial virulence, promote resistance to environmental stressors, and contribute to phage resistance via Superinfection exclusion (Sie) proteins (25, 28). These proteins prevent further infections by the same or closely related phages, inhibiting phage genome entry into bacterial cell (29).

*Salmonella* phages use LPS to enter host cells and establish a successful infection. Therefore, as part of the Sie mechanism, *Salmonella* may acquire prophage genes that modify the LPS structure (30).

Based on these considerations, we tested the potential of R^R^ to reduce the LPS reactogenicity through Lipid A modification, proposing it as an alternative approach for developing LPS-based vaccines against *Salmonella* infections. We found that the acquisition of the phage-resistant phenotype induces metabolic and physiological changes in R^R^, which alter the host-pathogen interaction by remodeling the LPS acylation pattern. Furthermore, we demonstrated that Lipid A modification in R^R^-LPS impairs the TLR4 signaling, weakening the innate immune response while mediating early protection in BALB/C mice infected with *S.* Rissen.

In conclusion, our findings offer a promising strategy for developing effective LPS-based vaccine to prevent *Salmonella* infection and may pave the way for the development of broad-spectrum vaccines against other Gram-negative bacteria.

## Materials and methods

### Ethics statement

All mouse experiments were carried out in accordance with the guidelines for the Care and Use of Laboratory Animals of the European Community (Legislative Decree n. 116/92). The Animal Use Protocol (AUP, Number: 86/609/CEE) was approved by the Committee on the Ethics of Animal Experiments of the University of Naples and authorized from the Italian Ministry of Health.

### *Salmonella* Rissen strains and culture conditions

The bacteriophage-sensitive *S. enterica ssp. enterica* serovar Rissen (R^W^) was isolated from food products and characterized by Istituto Zooprofilattico Sperimentale del Mezzogiorno (Portici, Naples, Italy), using standard conventional methods (31). The bacteriophage-resistant *S.* Rissen (R^R^), instead, was obtained from the R^W^ strain following selection for resistance to phage Ф1, as described by Papaianni et al. (25). Both R^R^ and R^W^ strains were grown in Luria-Bertani (LB) medium (ThermoFisher Scientific, Waltham, Massachusetts, USA) at 37°C, with shaking at 200 rpm.

### Phage Ф1 isolation

As previously reported by Papaianni et al. (25), bacteriophage Ф1 was isolated from the R^W^ strain upon induction with mitomycin C. Briefly, the overnight culture of the R^W^ strain was inoculated in 5 mL of fresh LB medium (2 x 10^8^ CFU/5 mL) containing 1 μg/mL mitomycin C (Merck, Darmstadt, Germany) and incubated for 1 hour at 37°C with shaking. After incubation, the induced culture was centrifugated at 5,700 x g for 10 minutes. The supernatant was collected and stored at +4°C, while pellet was resuspended in 5 mL of fresh LB medium and incubated for further 4 hours at 37°C with shaking. After 4 hours, lysate was centrifugated again and the supernatant collected. Supernatants from both the centrifugations were pooled and filtered by 0.22 μm filters (Millipore, Darmstadt, Germany) (25).

The enumeration of phage particles, expressed as plaques forming colony (PFU/mL), was determined by performing the Double Agar Layer (DAL) method (32). Plaques were re-isolated, propagated and stored in SM buffer (NaCl 100 mM, Tris-HCl 50 mM, MgSO_4_ 8 mM; pH 7.5) at + 4°C, or in liquid nitrogen with DMSO 5% (v/v) to avoid loss of phage titer (33).

### Bacteriophage-resistant and sensitive *Salmonella* Rissen genome comparison

Genotyping differences between bacteriophage-resistant and sensitive *S.* Rissen strains were explored using circular genome visualization methods. Protein Basic Local Alignment Search Tool (BLASTp) was used to match the sequence similarities between the SieB protein of *Salmonella* Typhimurium phage 22 (GenBank: AF217253, Protein ID: AAF75022) and the Superinfection exclusion protein of *S.* Rissen phage 29485 (Ф1, GenBank: KY709687, Protein ID: ARB10858). Genome visualization of *SieB* gene in both *S.* Rissen strains was performed using BLAST Ring Image Generator (BRIG) software (34) (v0.95). Both R^R^ and R^W^ complete genome sequence were reported by Papaianni et al. (25). *S.* Rissen phage 29485 was selected as reference genome. The raw sequence data are available via the European Nucleotide Archive (ENA; http://www.ebi.ac.uk/ena) and are accessible through the accession number PRJEB69481. The genome assembly and annotation of the RR strain can be accessed via European Nucleotide Archive under the following accession number GCA_963679805.

### Cell culture

The human gastric adenocarcinoma cell line AGS was kindly provided by Prof. Alessandra Tosco from the University of Salerno (Fisciano, SA, Italy). Cells were grown in Dulbecco’s Modified Eagle Medium (DMEM) high glucose (Microtech), supplemented with 10% fetal bovine serum (FBS; Microtech), 1% penicillin/streptomycin (Microtech) and 1% L-glutamine (Microtech) and maintained at 37°C, in a humidified atmosphere of 5% CO_2_. The cell line was authenticated through the short tandem repeat (STR) analysis and periodically tested for mycoplasma contamination.

### Superinfection exclusion gene transcriptional changes

To better investigate genome differences between the bacteriophage-resistant and sensitive *Salmonella* Rissen strains, due to prophage gene integration, the presence of the *SieB* gene was analyzed by end-point PCR and electrophoretic run on an automated qiaxcel instrument (Qiagen, Hilden, Germany) as described by Capparelli et al. (26). In addition, transcriptional changes in *SieB* gene were examined by real-time quantitative PCR experiment (RT-qPCR), using StepOne (Applied Biosystem) Real-Time PCR system. As reported by Capparelli et al., AGS cells were infected with R^R^ or R^W^ strains for 2, 4 and 6 hours and *SieB* gene expression level was evaluated by qRT-PCR, following the protocol described by Capparelli et al. (26). The primers for detecting *SieB* gene were: reverse primer 5’-CAAACAAATCCCGAACGACT-3’; forward primer 5’-ATGGTGGCAGGAGTTAATGC-3’.

### LPSs isolation and chemical analyses

Dried cells of R^R^ and R^W^ *S.* Rissen strains were extracted by PCP (phenol:chloroform:petroleum ether 2:5:8) and by water/phenol method as already reported (35).

PolyAcrylamide Gel Electrophoresis (PAGE) was performed using the system of Laemmli (36) with sodium deoxycholate (DOC) as detergent. Purified LPS from *S.* Rissen R^R^ and R^W^ and the LPS from *Escherichia coli* O111:B4 were prepared at the concentration of 1 mg/mL, and denaturated with sample buffer (2% DOC and 60 mM Tris/HCl pH 6.8, 25% glycerol, 14.4 mM 2-mercaptoethanol, and 0.1% bromophenol blue). Then, 8 μL of each sample was loaded on a 14% DOC gel with a 5% stacking gel and separated using a mini-PROTEAN electrophoresis instrument (Bio-Rad Laboratories). The electrophoresis was conducted at a constant amperage of 30 mA. The gel was fixed in an aqueous solution of 40% ethanol and 5% acetic acid, and finally visualized after silver nitrate staining (37). Precision Plus Protein Dual Color Standards (4 μL, MW size range 10–250 kDa) were used for molecular weight estimation. Glycosyl and fatty acids composition were obtained after derivatization as acetylated methyl glycosides (AMGs) and fatty acid methyl esters (FAMEs), as already described (38).

The linkage positions of the monosaccharides were determined by GC-MS analysis of the partially methylated alditol acetates (PMAAs). Samples (1 mg) were methylated with CH_3_I (100 µL) and NaOH powder in DMSO (300 µL) for 20 h (39). The products were totally hydrolyzed with 2 M TFA at 120°C for 2 h, reduced with NaBD_4_, and acetylated with Ac_2_O and pyridine (50 µL each, 100°C for 30 min).

All the derivatives were analyzed on Agilent Technologies gas chromatograph 7820A equipped with a mass selective detector 5977B and an HP-5 capillary column (Agilent, 30 m × 0.25 mm i.d.; flow rate, 1 mL/min). AMGs and FAMEs analyses were performed using different temperature programs. In detail, the AMGs analysis started at 140°C for 3 min, followed by an increase from 140°C to 240°C at 3°C/min; while the FAMEs analysis started at 140°C for 3 min, followed by an increase from 140°C to 280°C at 10°C/min. PMAA analysis was performed using a temperature program that began at 90°C for 1 minute, then increased from 90°C to 140°C at 25°C/min, 140°C to 200°C at 5°C/min, and 200°C to 280°C at 10°C/min, with a final hold at 280°C for 10 minutes.

### OPSs isolation, purification, and structural characterization

The supernatants obtained after mild acid hydrolysis of the entire LPSs (19.5 mg and 15 mg from R^R^-LPS and R^W^-LPS, respectively) were fractionated on a Biogel P-10 column (Biorad, 0.75 x 112 cm) eluted with water. The collected fractions were pooled, freeze-dried, and analyzed by ^1^H NMR spectroscopy.

The molecular weight determination for OPSs was obtained through a calibration curve by using an Agilent 1100 HPLC system, with a TSK G-5000 PWXL size exclusion column (30 cm × 7.8 mm) equilibrated with NH_4_HCO_3_ 50 mM as eluent (flow = 0.7 mL/min). The column was calibrated by injecting dextran standards (50 μL of a 1 mg/mL solution) of known molecular weight (12, 50, 150, and 670 kDa). The eluate was monitored with a refractive index detector (40).

### Lipid A isolation and characterization

The Lipid A fraction was obtained from crude LPSs of both R^R^ and R^W^ *S.* Rissen by mild acid hydrolysis, as reported (38). After centrifugation at 3000 rpm for 15 min, the supernatants were removed and the pellet suspended in water and freeze-dried (3 mg and 4mg, for R^R^ and R^W^ respectively). MALDI TOF mass spectrometry was performed in the reflectron negative ions mode using a ABSCIEX TOF/TOF™5800 (AB SCIEX, Darmstadt, Germany) instrument, equipped with an Nd: YLF laser with a λ of 345 nm, and a delayed extraction technology. A solution of 2,4,6-trihydroxyacetophenone (THAP) in MeOH:H_2_O (v/v 1:1, 10 mg/mL) was used as the matrix. After desalting on a Dowex 50WX8 (H^+^ form) the samples with a concentration of 2 mg mL^-1^ were dissolved using CHCl_3_:MeOH (v/v 2:1). The matrix solution (0.5 µL) and Lipid A solutions (0.5 µL) were spotted on a MALDI plate. Each spectrum was a result of 2000 laser shots. The spectra were calibrated by using an ABSciex calibration mixture, and processed under computer control by using the Data Explorer software (v0.2.0) (41). Fatty acids analyses were obtained as already described (38). In few words, 0.5 mg of each sample was treated with 1 mL of 1.25 M HCl/CH_3_OH for 20 h at 80°C. The crude mixtures were extracted twice with hexane, and injected into GC-MS. The analyses were performed on Agilent Technologies gas chromatograph 7820A equipped with a mass selective detector 5977B and an HP-5 capillary column (Agilent, 30 m × 0.25 mm i.d.; flow rate, 1 mL/min). A temperature program starting at 140°C for 3 min and followed by an increase from 140°C to 280°C at 10°C/min was used for fatty acid analyses.

### LPS purification for biological studies

LPS isolated from both R^R^ and R^W^ (38) were purified according to Stephens et al. (42). Briefly, LPSs were resuspended in endotoxin-free water containing triethylamine (TEA; 0.2%), deoxycholate (0.5%) and water-saturated phenol (500 μL). The obtained solution was vortexed for 5 minutes, incubated on ice for 5 minutes and then centrifugated (10,000 g for 2 minutes at 4°C). The upper phase (aqueous) was transferred into a fresh tube, supplemented with 75% ethanol and 30 mM sodium acetate, precipitated for 1 hour at -20°C and lastly resuspended in TEA.

### LPS conjugation for mice immunization

The R^R^ and R^W^-LPS were coupled to Rat Serum Albumin (RSA; purchased from Sigma Aldrich) using 1-ethyl-3-(3-dimethylaminopropyl) carbodiimide (EDC), following the protocol described by Masoud, 2007 (43). Briefly, purified LPSs (10 mg) were dissolved in sterile distilled water (1 mL), and the solution was mixed with 500 μL RSA (10 mg/ml), in the presence of EDC (40 mg). The mixture was stirred on an automatic shaker at room temperature for 3 h. After mixing, the pH was adjusted to 7 and the mixture was centrifuged (1000 rpm for 10 minutes). The supernatants were purified by using a Sephadex G-75 column and eluted with phosphate buffered saline (PBS). Eluates containing both LPS and protein were pooled and dialyzed against distilled water at 48°C, and finally lyophilized.

### Bacterial growth culture preparation for metabolomics analysis

R^W^ and R^R^ strains were obtained as indicated above (see the *S.* Rissen strains and culture conditions paragraph). R^W^Φ1- and R^S^Φ1- strains were derived from the R^W^ and R^R^ strains as described by Papaianni et al. (25). All strains were grown in LB medium and centrifugated when bacteria reached the exponential growth phase and the same cell density. After centrifugation, the pellet was discarded, and the supernatant filtered by 0.22 μm filters (Millipore, Darmstadt, Germany). Sterile supernatants were quenched in liquid nitrogen for 10 minutes - in order to stop metabolic activities in the filtrates - and stored at -80°C, until NMR analysis. Ten biological replicates of growth culture of each strain were prepared. Uninoculated growth medium was used as negative control.

### NMR sample preparation

To achieve the NMR-based footprinting metabolomics analysis, growth media from R^R^, R^W^, R^S^Φ1- and R^W^Φ1- were rapidly defrosted and 630 μL of each fluid sample were pipetted into eppendorfs containing 70 µL of D_2_O, containing sodium 3-(trimethylsilyl)-2,2,3,3-tetradeuteropropionate (TSP) 0.1 mM (for chemical shift reference), and sodium azide 3 mM as antimicrobial agent, reaching 700 μL of total volume.

### NMR spectra acquisition

NMR spectra were acquired as described by Papaianni et al. (44). In detail, all NMR experiments were performed on a Bruker Avance III-600 MHz spectrometer (Bruker BioSpin GmbH, Rheinstetten, Germany), equipped with a refrigerated autosampler and a TCI CryoProbe™ fitted with a gradient along the Z-axis, at a probe temperature of 300K (27°C). All spectra were recorded in H_2_O 90%:D_2_O 10% with 0.1 mM of TSP. ^1^H-NMR spectra were acquired with water suppression using excitation sculpting with gradience sequence (45). Spectra were acquired for 32 scans, with an acquisition time of 5.2 s, a relaxation delay of 2 s, a pulse width of 8.000 µs, receiver gain of 101, dwell time of 61 μs, 4 dummy scans and a spectral width of 13.0180 ppm. Two-dimensional homonuclear (^1^H-^1^H; TOCSY) spectra were acquired with water suppression using excitation sculpting with gradients, DIPSI-2 sequence (46). Mixing time of 100 ms was applied for TOCSY experiments. TOCSY experiments were acquired with 256 FIDs of 2048 complex points with 80 scans, a relaxation delay of 1 s, a pulse width of 8.000 µs, receiver gain of 101, dwell time of 64 μs, 32 dummy scans and a spectral width of 13.6582 ppm. All data were zero-filled to 4,096 points in both dimensions, and prior to Fourier transformation, a Lorentz- to-Gauss window with different parameters was applied for both t_1_ and t_2_ dimensions for all the experiments. The resonances of both 1D and homonuclear 2D spectra were referenced to internal sodium 3-(trimethylsilyl)-2,2,3,3-tetradeuteropropionate (0.1 mM; TSP) which was assumed to resonate at δ_H_ = 0.00 ppm. Two-dimensional heteronuclear (^1^H-^13^C; HSQC) spectra were recorded on the spectrometer operating at 150.90 MHz for ^13^C, using pre-saturation for water suppression (47, 48). HSQC resonances spectra were referenced to the lactate doublet (βCH_3_), assumed to resonate at 1.33 ppm for ^1^H and 20.76 ppm for ^13^C.

^1^H NMR spectra of the O-chains were recorded by using a Bruker 600 MHz spectrometer equipped with a cryo-probe, at 298K. The samples (3 mg) were dissolved in 550 μL of D_2_O and the spectra were calibrated with external acetone (δ_H_ = 2.225 ppm; δ_C_ = 31.45 ppm). Spectra were acquired for 32 scans, with an acquisition time of 4.58 s, dwell time of 70 µs, and spectral width of 11.90 ppm.

### NMR data analysis

To obtain bacteria growth culture datasets, a spectral area ranging from 9.50 to 0.50 ppm was selected. Prior the data analysis, each proton spectrum was manually phased, and the baseline corrected using Buker Tospin software (v.3.6.3). Successively, they were automatically segmented into integrated regions (buckets) of 0.02 ppm, using the AMIX 3.6 package (Bruker Biospin, Germany). The residual water resonance region (5.00-4.58 ppm) was excluded, and the binned regions were normalized to the total spectrum area. Multivariate statistical data analysis was applied to each dataset, to differentiate culture medium profiles of bacteriophage-sensitive or resistant *S.* Rissen, before and after lysogenic Ф1 excision (R^W^ or R^R^ and R^W^Φ1- or R^S^Φ1-, respectively), through NMR spectra, according to their different metabolic content. Therefore, each integrated dataset – containing information on metabolite concentration - was exported to excel and the derived data matrix imported into SIMCA-P17 package (Umetrics, Umea, Sweden) where unsupervised Principal Component Analysis (PCA), supervised Partial Least Squares Discriminant Analysis (PLS-DA) and Orthogonal Partial Least Squares Discriminant Analysis (OPLS-DA) analyses were performed. PCA was first applied to check outliers and uncover initial trends within the dataset by investigating the systematic variation in the data matrix, in order to identify trends and clusters. Once assessed data homogeneity, PLS-DA or OPLS-DA discriminant analysis was employed to improve group discrimination. The performance of each elaborated model was evaluated via the parameters R2 and Q2 (PCA: R2 = 0.879, Q2 = 0.769; OPLS-DA: R2 = 0.99, Q2 = 0.873), indicating the goodness of fit and the goodness of prediction, respectively. NMR variables with |pcorr| ≥ 0.7, VIP >1 (Variable Importance in the Projection) were then considered for metabolite identification and univariate statistical analysis. Metabolites were identified through the chemical shift, according to both the literature (49) and metabolomics databases, such as the Human Metabolome Database (HMDB). Statistical significance was determined by one-way analysis of variance (ANOVA) followed by Bonferroni *post hoc* correction. *p* values < 0.05 were considered statistically significant.

### Hemolytic activity

The susceptibility of erythrocytes to R^R^ or R^W^-LPS was evaluated by performing the hemolysis assay on fresh human blood. Erythrocytes were isolated from blood of healthy donors (n=9), in accordance with the principles of good clinical practices reported in the Declaration of Helsinki and under the approval of the Ethics Committee of Villa Betania Hospital. Here, blood samples were obtained from the arm’s peripheral vein and collected in heparin tubes. An informed consent was obtained from all participants.

As reported by Greco et al. (50), whole blood was centrifuged at 1,500 x g for 5 minutes and the supernatant (plasma) discarded. Cell pellet, instead, was washed with PBS and gently shacked. These operations were repeated 5 times. Aliquots of 5 x 10^7^/mL red blood cells were resuspended in PBS containing different concentrations of R^R^ or R^W^-LPS (from 1 to 500 μg/mL), incubated at 37°C for 1 hour and centrifuged at 1,500 x g for 5 minutes. The supernatant was removed, and the released hemoglobin measured at 405 nm, using a plate reader. The percentage of hemolysis was calculated as the ratio of the untreated control.

### Animal experiments

Animal experiments were carried out on female BALB/c mice aged 8 to 10 weeks - purchased from Charles River Laboratories (Wilmington, Massachusetts, USA) - at the animal facility of the University of Naples “Federico II”. Animal were housed in static microisolator cages, in order to handle them aseptically, and fed with sterile food and beverage. Mice were randomly assigned to the treatment groups. The sample group allocation was not blinded to the investigators.

Six mice per group were infected intraperitoneally with 100 μL of sterile phosphate-buffered saline (PBS), containing grown doses (10^3^, 10^5^, 10^7^ and 10^9^ CFU/animal) of R^R^ or R^W^ strains. Control group, instead, received 100 μL of PBS only. Mice were observed for 21 days, in order to evaluate clinical manifestations and death. Lethal Dose-50 (LD_50_) was calculated according to the Reed and Muench method (51).

LPS purified from both R^R^ and R^W^ strains was administrated intraperitoneally in a dose responsible for endotoxemia (20 mg/Kg body weight) (52) and mice (6 per group) were observed 2, 8 and 24 hours after the LPS injection, in order to evaluate body temperature and weight. As for viable bacteria, LPS was dissolved in PBS (100 μL per animal) and the control group was injected with PBS only. Mice that lost more than 20% of their starting body weight or decreased their starting body temperature more than 4°C were euthanized.

### Cytokine measurement by ELISA assay

Blood samples were collected from mice via tail vein bleeding, after application of a local anesthetic. Blood was collected 2-4-6-8- and 24-hours following R^R^ or R^W^-derived LPS administration and was immediately centrifugated at 3,000 g for 10 minutes at + 4°C. Blood samples collected from mice injected with sterile PBS were used as control. After centrifugation, serum was separated and frozen at - 80°C, until cytokine measurement was performed. The concentration of IL-6, IL-10, TNF-α, G-CSF and IFN-γ cytokines was determined by multiplex ELISA assay, using the Bio-Plex pro-mouse cytokine 23-plex assay (Bio-Rad), according to the manufacturer’s recommendations. Briefly, samples were diluted 1:4 with sample diluent and assayed as previously described by Verrillo et al. (53). Data were collected using the Bio-Plex 200 System (Bio-Rad) and analyzed by Bio-Plex Manager™ software. In addition, the concentration of IFN-β, was determined by the single target ELISA assay, using the mouse IFN-β ELISA kit (Elabscience), according to the manufacturer’s instructions. Samples were read using the 96-well plate reader (NeoBiotech). Cytokines were measured at different times and their concentration was determined based on the automatically calculated standard curve.

### Immunization

Mice were divided into three groups, each group containing 6 mice. The first group was injected intraperitoneally with the R^R^-entire LPS, while the second one with the R^W^-entire LPS. Both the antigens were extracted, purified and conjugated with Rat Serum Albumin (RSA) as reported above (“LPS purification for biological studies” and “LPS conjugation for mice immunization”). For the immunization, they were dissolved in sterile PBS (pH=7.4) and administrated twice at two-weeks interval at the final concentration of 10 μg (100 μL). The third group (control group) was injected with sterile PBS only (100 μL). On day 21, a secondary booster was administrated. Blood samples from the tail vein and feces were collected on day 4, 9, 14, 21 and 28 to determine the adaptive immune response and evaluate the cross-protective ability conferred by the R^R^-LPS immunization, as described below.

### Antibody response: IgG, IgA and IgM detection

To investigate the levels of adaptive immune response following animal immunization, the levels of IgG, IgM and IgA were detected. Serum was separated from whole blood by centrifugation (3,000 g, 10 minutes at + 4°C) and used to measure LPS-specific IgG and IgM, while feces were resuspended in protein extraction buffer (100 μL/10 mg), vortexed for 20 minutes and centrifugated 13,000 x g for 10 minutes. Supernatants were collected and used to detect LPS-specific IgA. First, the total antibody level for each Ig was detected using the mouse IgG Elisa kit (Abcam, Cambridge, United Kingdom), IgM ELISA kit (Abcam, Cambridge, United Kingdom) and IgA (Bethyl Laboratories, Montgomery, Texas, USA), according to the manufacturer’s recommendations. Successively, the anti-LPS IgG, IgM or IgA titer was quantified using LPS coated microplates, according to the standard indirect ELISA protocol (54). Briefly, 96-well plate was coated with R^R^ or R^W^ purified LPS (5 µg/mL) in 100 µL/well coating buffer (Na_2_CO_3_ 0.64 g and NaHCO_3_ 3.7 _g_) and incubated overnight at + 4°C. The next day, wells were blocked with 200 µL/well of wash-buffer containing 2% bovine serum albumin (BSA) and the plate was incubated for 1h at 37°C. Sera or fecal supernatants were diluted 1:100 in blocking buffer (100 µL/well) and incubated at 37°C for 1 h. Following incubation, 100 µL of the HRP-conjugated goat anti-mouse IgG (1:2,000; Invitrogen), IgA (1:10,000; Abcam) or IgM (1:1,000; Invitrogen) were added to each well and incubated at 37°C for 30 min. Finally, the plate was filled with 100 µL/well of the indicator substrate (TMB; 1 mg/mL to each well) and incubated at room temperature for 10 min in the dark. After incubation the enzymatic reaction was stopped by adding 100 µL/well of stop solution (1M sulphuric acid) and 15-30 minutes later the optical density (OD) was recorded at 450 nm with an automatic ELISA reader (NeoBiotech). The assay was performed in triplicate and each incubation period was followed by multiple washings using PBS containing Tween 20 (0.05%). The standard curve was used to accurately determine the concentration of target LPS-immunoglobulins.

### Evaluation of the R^R^-LPS immunization efficacy

The efficacy of the R^R^-LPS immunization was evaluated by using a mouse model of salmonellosis producing disseminated infection after intraperitoneal injection of the bacteriophage-sensitive *Salmonella* Rissen *(*R^W^). In detail, mice immunized as detailed above (see the “Immunization” paragraph) were infected with the R^W^ strain (10^9^ CFU/100 μL) two weeks after the last immunization via the intraperitoneal route and the bacterial burden in the spleens was determined. Therefore, mice were sacrificed 3 days post-infection and spleens were aseptically removed. Spleens (1 g) were homogenized in 1 mL of phosphate buffer saline (PBS) solution and serial dilutions were cultured on BHI agar for 24 hours, at 37°C. The next day, colony were counted, and results were expressed as colony forming unit (CFU) per gram of tissue (CFU/spleen).

Additionally, the protective efficacy of the R^R^-LPS was also evaluated upon a single immunization. Mice (n=13 per group) were immunized intraperitoneally with pure conjugated LPS (10 μg dissolved in PBS 100 μL) isolated from the R^R^ or R^W^ strains or with vehicle (PBS 100 μL; control group). Two weeks post-immunization, each group was infected with the R^W^ strain (10^9^ CFU/100 μL) via the intraperitoneal route and the bacterial burden in the spleens was determined 3 days post-infection.

### Antibody bactericidal activity

To investigate the role of anti-R^R^-LPS antibodies in imparting immune protection against *S.* Rissen infection, the bactericidal assay was performed. Mice were immunized as described above and four days after the last immunization, blood samples were collected, and serum separated by centrifugation. Polyclonal antibodies were purified by affinity chromatography, using protein A/G agarose beads as previously described (55, 56). 45 μL of antibody solution, antibody solution plus complement (BioMerieux, France), complement or LB broth (for control group) were mixed with 5 μL of viable R^W^ (final bacterial concentration 1 x 10^6^ CFU/mL) in a 96-well plate and incubated at 37°C for 3 hours. After incubation, the absorbance at 600 nm was measured using a microplate reader. Data are reported as Log_10_ of CFU/mL.

### Analysis of the cross-protective ability of anti-*R^R^*-LPS antibodies

To evaluate whether the R^R^-LPS immunization could confer cross-protection, serum samples from mice immunized with the R^R^-LPS were tested for antibody reactivity against three different serovars of *Salmonella* (*S.* Choleraesuis*, S.* Infantis*, S.* Newport) by the agglutination test.

Mice were immunized as described above and four days following the last immunization, blood samples were collected, and the serum was separated by centrifugation (3,000 g, 10 minutes at + 4°C). Serum samples were used to measure IgG levels by the LPS-specific ELISA assay and animals giving the highest level of antibodies were selected for serum harvest. For the agglutination test, colonies of the selected *Salmonellae* were mixed with 100 μL of serum (undiluted or diluted 1:50 or 1:100 with sterile PBS) or with sterile PBS only (negative control). *S.* Rissen was used as positive control. The presence of cross-reaction was determined by observing the development of visible clumping of bacterial cells within 3 minutes.

### Evaluation of the anti-R^R^-LPS antibodies target determinant to *S.* Rissen LPS

To explore the target determinant of the anti-R^R^-LPS antibodies to *S.* Rissen LPS, an Indirect ELISA assay was performed. We tested the antibody reactivity against both LPS and O-antigen from the R^R^ and R^W^ strains using serum samples from mice immunized with the R^R^-LPS, as reported above. Briefly, a 96-well plate was coated with O-antigen (5 µg/mL) or LPS (5 µg/mL) in 100 µL/well coating buffer (Na_2_CO_3_ 0.64 g and NaHCO_3_ 3.7 g) for 16 h at 4°C. Sera were diluted 1:100 in 100 µL/well of wash-buffer containing 2% bovine serum albumin (BSA) and incubated at 37°C for 1 h. Following incubation, 100 µL of the HRP-conjugated goat anti-mouse IgG diluted 1:2,000 (Invitrogen) was added to each well and incubated at 37°C for 30 min. Finally, the plate was filled with 100 µL/well of the indicator substrate (TMB; 1 mg/mL to each well) and incubated at room temperature for 10 min in the dark. After incubation the enzymatic reaction was stopped by adding 100 µL/well of stop solution (1M sulphuric acid) and 15-30 minutes later the optical density (OD) was recorded at 450 nm with an automatic ELISA reader (NeoBiotech). The assay was performed in triplicate and each incubation period was followed by multiple washings using PBS containing Tween 20 (0.05%).

### Statistical analysis

Statistical analysis was conducted using GraphPad Prism 9.0 software (San Diego, CA, USA). One-way ANOVA, followed by either Turkey or Bonferroni *post-hoc* correction, was utilized to compare two or more experimental groups. For simultaneous comparison of multiple groups with two or more factors, we used two-way ANOVA followed by Bonferroni *post-hoc* test. The Log-rank test was employed to compare Kaplan–Meier survival curves. A minimum of n = 6 mice per group was included, and no formal randomization procedure was implemented. The reported values represent the means ± SD of two or three biological replicates, as indicated in figure legends. A *p*-value of < 0.05 was considered statistically significant.

## Results

### Prophage genes promote the expression of a phage-resistance phenotype

**Figure 1:**
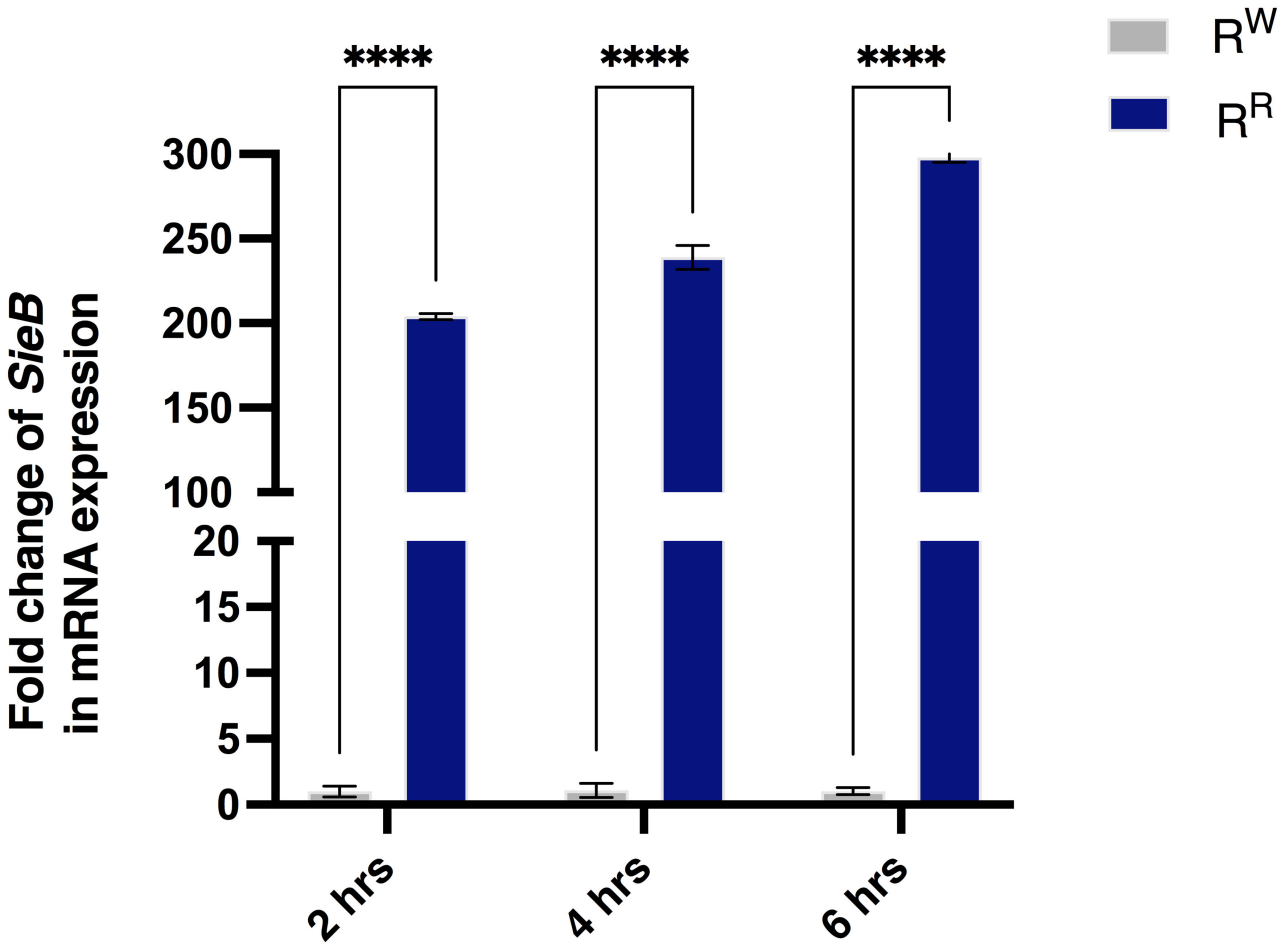
The R^R^ strain expresses the prophage *SieB* gene. Analysis of mRNA expression levels displayed increased levels of *SieB* gene in the R^R^ strain - following AGS cell line infection - compared to R^W^ strain. Results are reported as graph bars and represent mean ± SD of three biological replicates from two independent experiments. Two-way ANOVA followed by Turkey *post-hoc* correction was used to compare the *SieB* gene expression between R^R^ and/or R^W^ groups, according to different time points. ****p < 0.0001.

The property of prophages to mediate phenotypic changes in bacterial host is widely known. Our previous studies demonstrated significant differences in virulence profiles between R^W^ and R^R^ (26). Importantly, R^R^ exhibited higher expression levels of prophage-related virulence genes (*sspH1, sodC1, gtgE, grvA*, and *gogB*) than R^W^ (26). In the present study, we also observed transcriptional changes in the *Superinfection exclusion* (*SieB*) gene, which affects the bacterial membrane integrity and inhibits phage infection (57). As expected, R^R^ expressed *SieB* at a level 300-fold higher than R^W^ (**Figure 1**), in a time-dependent manner.

To further investigate the genetic diversity between R^R^ and R^W^, a genome comparison analysis, using the prophage Φ1 genome as a reference (*Salmonella* phage 29485, GenBank: KY709687), was performed. The circle plot revealed the insertion of the prophage derived *SieB* gene into the R^R^ genome (**Supplementary Figure S1**). The evidence that the gene under investigation was *SieB*, was provided by the Basic Local Alignment Search Toll (BLAST, https://blast.ncbi.nlm.nih.gov/Blast.cgi), showing robust sequence similarity between *Salmonella* phage 29485 superinfection exclusion protein (Protein Id: ARB10858) and *Salmonella* phage P22 SieB protein (Protein Id: AAF75022, **Supplementary Figure S2**).

In conclusion, these results suggest that R^R^ expresses the *SieB* gene, which confers immunity against secondary phage attack and contributes to the phage-resistance phenotype, likely through modifications of cell membrane elements, such as LPS (28). In addition, these results, along with previous reported results (26), prompted us to investigate whether the prophage-related virulence genes overexpressed by R^R^ confer advantages to the host response against *Salmonella*.

### Phage-resistance alters the LPS structure by inducing the production of under-acylated Lipid A

To investigate whether phage-resistance alters the LPS structure and to assess the effects of these modifications on bacterial virulence and host immune responses, the chemical structure of the Lipid A and O-antigen portions was examined.

Dried cells from both R^R^ and R^W^ strains were extracted as previously described (35). Both R^R^ and R^W^ produced smooth LPS (**Supplementary Figure S3A**). Additionally, GC-MS chromatograms of the purified O-chain polysaccharides (OPSs) revealed no differences in the sugar content of the O-antigen between the R^R^ and R^W^ strains (**Supplementary Figure S3B**). Methylation analysis further indicated the presence of 2-substituted mannose units (2-Man), 2,3-disubstituted mannoses (2,3-Man), 3-substituted glucosamine (3-GlcN) and terminal non reducing glucose (t-Glc) in the O-chains of both strains (**Supplementary Table S1**). Finally, in line with chemical analyses, ^1^H NMR spectra revealed an identical structure for the purified OPSs (**Supplementary Figure S3C**). In contrast, differences in the Lipid A portion were detected.

**Figure 2:**
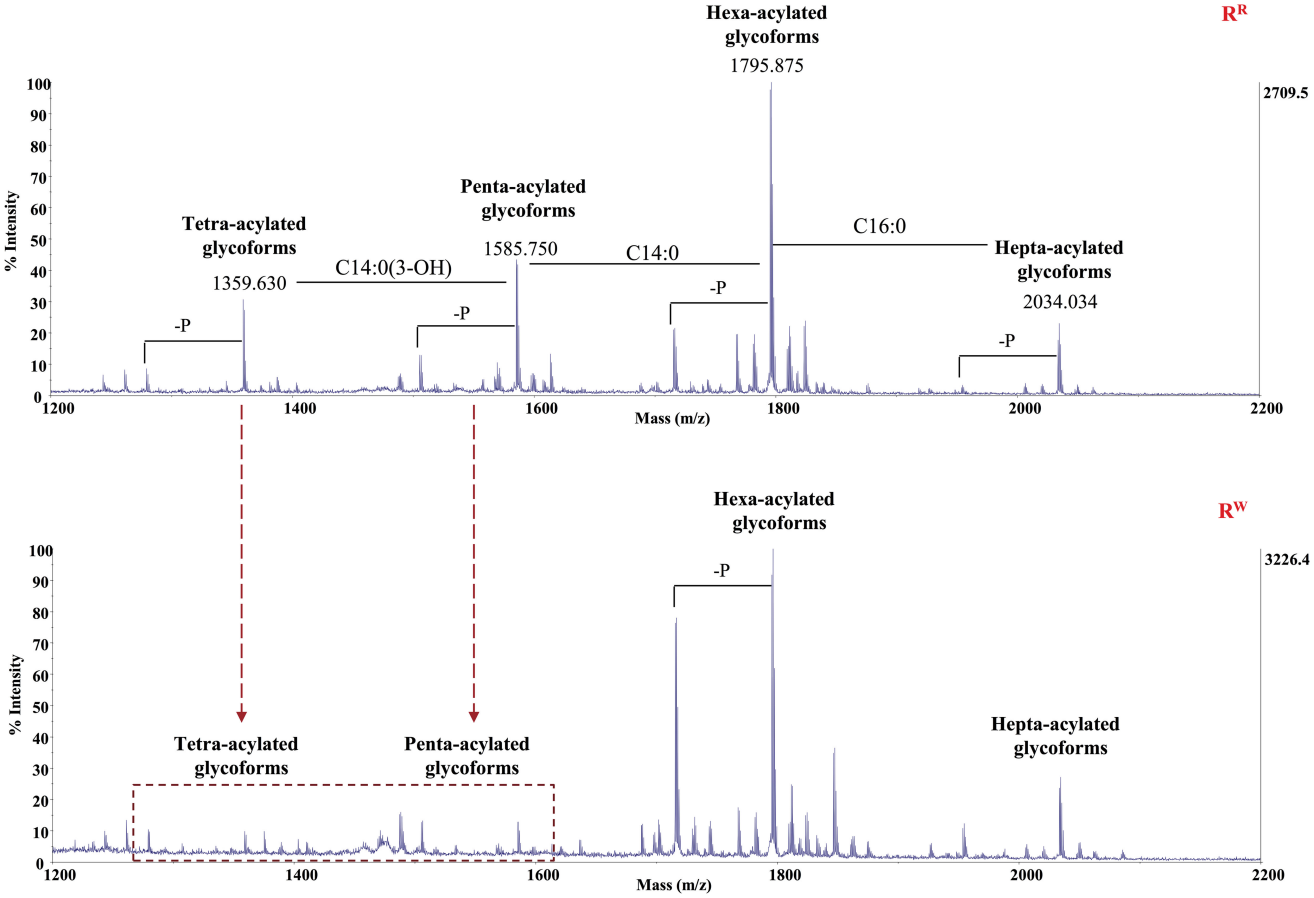
Reflectron negative MALDI-TOF MS spectra of *S.* Rissen R^R^ and R^W^ Lipid A.

Pure Lipid A was analyzed through chemical techniques and mass spectrometry. GC-MS analysis of the fatty acid methyl esters (FAMEs) disclosed the presence of mainly C12:0, C14:0, C16:0, and C14:0(3-OH), in both the Lipid As. In addition, Lipid A from the R^W^ strain was found to contain C14:0(2-OH). The Lipid A samples were analyzed by negative ions MALDI- TOF in reflectron mode. The acquired spectrum of Lipid A from R^R^ revealed four clusters of signals corresponding to glycoforms with distinct acyl patterns (**Figure 2**). More in detail, the primary signal at m/z 1795.875 (calculated [M-H]^-^ = 1796.229 Da) was assigned to the di-phosphorylated hexa-acylated glycoforms HexN_2_P_2_[C14:0(3-OH)]_4_(C14:0)(C12:0). The spectrum also showed the presence of a signal at m/z 2034.034 corresponding to the di-phosphorylated hepta-acylated species (calculated [M-H]^-^ =2034.462 Da), which differs from the hexa-acylated species for the additional presence of a C16:0 and, signals at m/z 1585.750 and 1359.630 corresponding to the di-phosphorylated penta- and tetra-acylated glycoforms, respectively. Finally, each cluster exhibited heterogeneity as proven by the occurrence of signals differing in 80 u, corresponding to the phosphate group, and in 14 u (–CH_2_– unit), which indicates Lipid A species differing in the length of their acyl chains.

In conclusion, these results suggest that R^R^ LPS contains highly variant Lipid A species, including di-phosphorylated hexa- and hepta-acylated species, along with under-acylated species (di- phosphorylated tetra- and penta-acylated variants). The same pattern was observed in the R^W^ LPS. Nevertheless, R^W^ LPS shows an extremely reduced presence of di-phosphorylated tetra- and penta-acylated species, compared to R^R^ LPS. It is well known that under-acylated variants of Lipid A compete with the hexa-acylated form for TLR4 activation, functioning as weak agonists of TLR4 signaling (58). Specifically, under-acylated Lipid A species destabilize the MD-2–TLR4 complex, leading to a reduced production of pro-inflammatory cytokines (59, 60). Based on these considerations, our findings suggest that R^R^-LPS is a weak stimulator of the host innate immune response and elicits a mitigated inflammatory response.

### Phage-resistance alters host metabolic profiles

**Figure 3:**
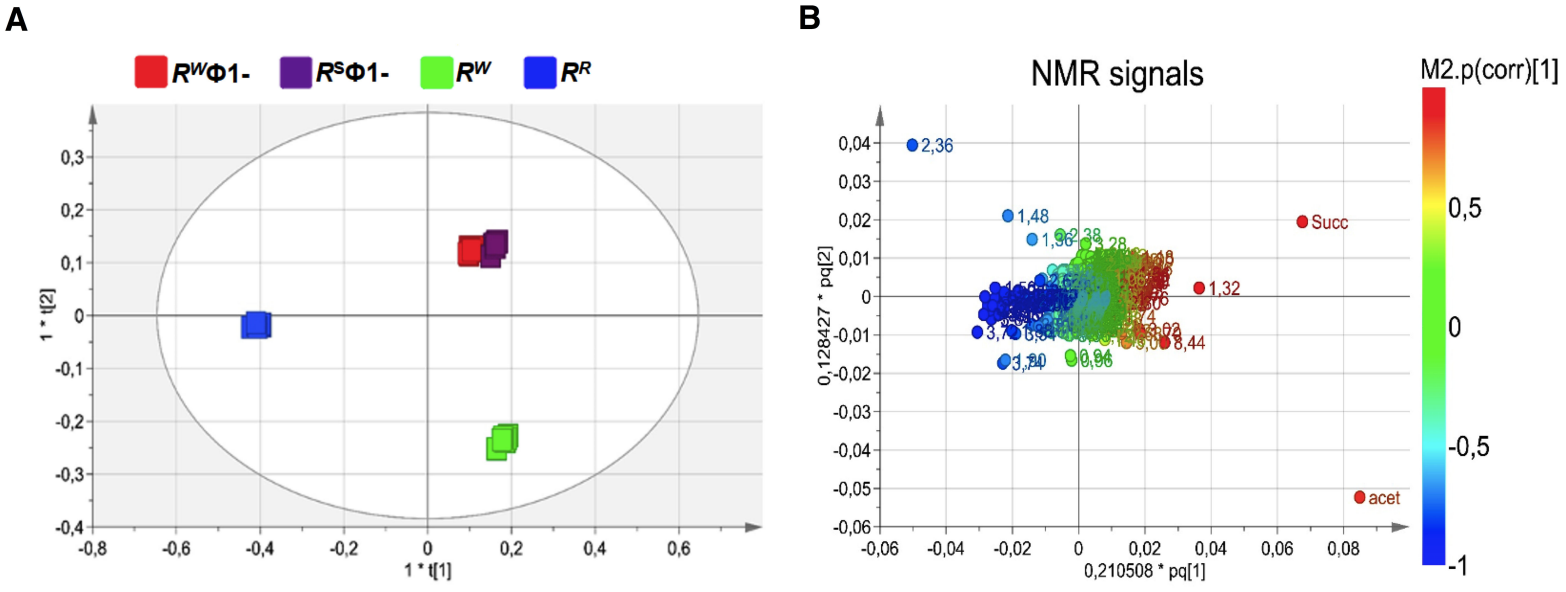
Orthogonal Projection to Latent Structure Discriminant Analysis (OPLS-DA) of bacteriophage-sensitive or resistant *S.* Rissen medium samples, before or after phage excision (R^W^; R^R^; R^S^Ф1-; R^W^Ф1-, respectively). **(A)** Scores plot showing a distinct separation of R^R^ group (blue squares) from R^W^, R^W^Ф1- and R^S^Ф1- groups (green, red and purple squares, respectively) and of R^W^Ф1- and R^S^Ф1- groups (red and purple squares, respectively) from the R^W^ group (green squares). **(B)** Loading plot indicating NMR variables (chemical shift) of metabolites responsible for between-classes separation, characterized by |p(corr)| value > 0.7.

**Figure 4:**
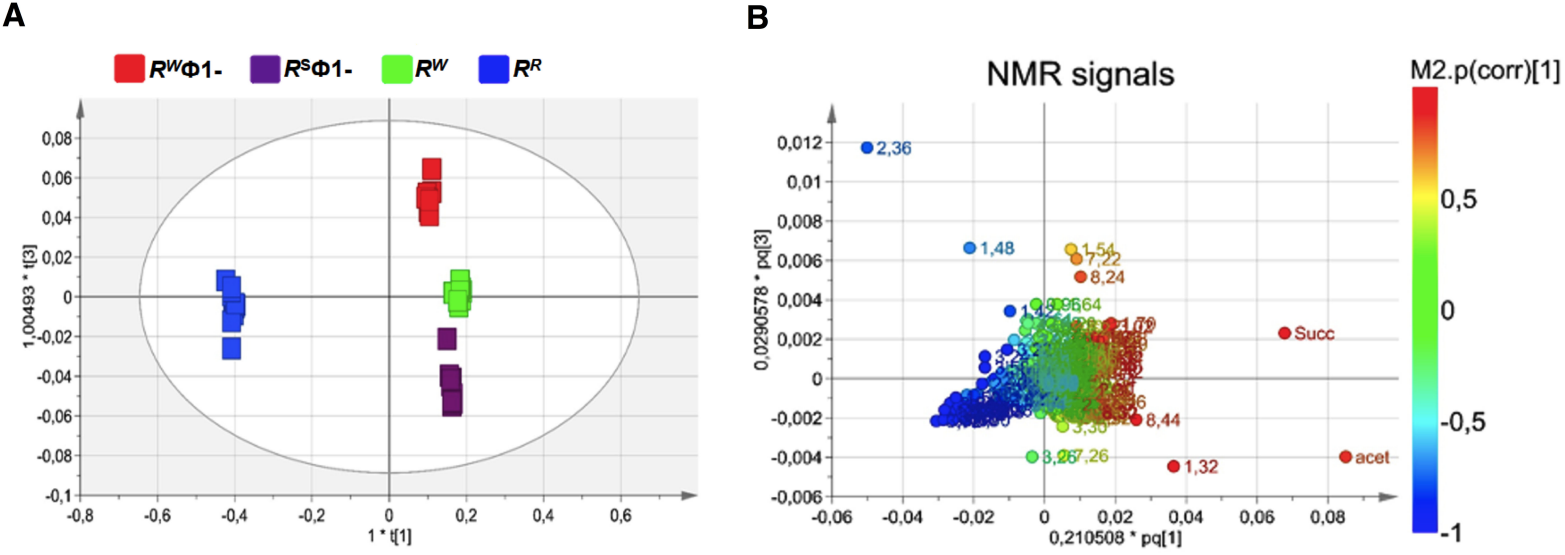
Orthogonal Projection to Latent Structure Discriminant Analysis (OPLS-DA) of bacteriophage-sensitive or resistant *S.* Rissen medium samples, before or after phage excision (R^W^; R^R^; R^S^Ф1-; R^W^Ф1-, respectively). **(A)** Scores plot showing a distinct separation of R^S^Ф1- group (purple squares) from R^W^Ф1- group (red squares). **(B)** Loading plot indicating NMR variables (chemical shift) of metabolites responsible for between-classes separation, characterized by |p(corr)| value> 0.7.

In order to investigate the physiology of R^R^ and its capability to adapt to various host conditions and evade host innate immune attack, basic insights into bacterial metabolism are fundamental. Culture supernatants from R^R^ or R^W^ *–* before and after lysogenic Ф1 excision (R^R^ or R^W^ and R^S^Ф1- or R^W^Ф1, respectively) *–* were compared by untargeted NMR-based metabolomics analysis. NMR spectra of growth medium from the wild-type R^W^ strain and the selected R^R^ mutant displayed extensive differences. Conversely, growth medium from R^W^ and R^R^, following phage excision (R^W^Ф1- and R^S^Ф1-, respectively) showed a considerable similarity in the NMR profiles (**Supplementary Figure S4**). NMR spectra were compared by performing multivariate analysis. The OPLS-DA scores plot revealed a clear separation of the R^R^ group (placed at negative coordinates of the first component t[1]) from R^W^, R^W^Ф1- and R^S^Ф1- groups (placed at positive coordinates of the first component t[1], **Figure 3A**). This result might be attributable to metabolic alterations associated with phage-resistance. The second component t[2], instead, revealed a distinct separation of R^W^Ф1- and R^S^Ф1- groups (placed at positive coordinates) from the R^W^ group (placed at negative coordinates), likely due to phage-specific changes in the metabolism of the bacteriophage-sensitive strain (**Figure 3A**). Based on the first two principal components (t[1] and t[2]), R^W^Ф1- and R^S^Ф1- groups were found closely related each other, forming a single cluster in the hotteling’s T2 plot (**Figure 3A**). A more evident separation between R^W^Ф1- and R^S^Ф1- groups was detected by the third component t[3], which positioned the R^W^Ф1- group at t[3] positive coordinates and the R^S^Ф1- group at t[3] negative coordinates (**Figure 4A**).

A total of 28 metabolites were identified as responsible for the above reported classes separation (**Supplementary Table 2**). In fact, significant differences in their concentration were detected. In detail, 14 amino acids and derivatives (arginine, lysine, isoleucine, proline, leucine, glutamate, methionine, aspartate, glycine, alanine, phenylalanine, tyrosine, N-acetyl-alanine and γ-aminobutyric acid), 1 sugar (glucose), the nucleic acid derivative hypoxanthine, the short chain fatty acid (SCFA) acetate, and 5 organic acid and derivatives (pyruvate hydrate, 5-aminopentanoic acid, lactate, succinate and formate) discriminated the R^W^ group from the R^R^ (*p* < 0.05). Interestingly, R^S^Ф1- and R^W^Ф1- groups did not display significant differences (*p* > 0.05) in the concentration of succinate, lactate, 5-aminopentanoic acid, formate, arginine, lysine, proline and phenylalanine. This result suggests that the different concentration observed in the growth medium from R^W^ and R^R^ is ascribable to metabolic changes induced by the presence of the phage Ф1 and specifically to endogenous phage genome elements responsible for phage-resistance.

Similarly, phage infection altered the expression levels of 3 amino acids and derivatives (betaine, threonine and pyroglutamate), 1 sugar (myo-inositol) and other metabolites (nicotinate and imidazole). These metabolites were present in different concentrations (*p* < 0.05) in the growth medium from R^W^Ф1- compared to R^W^. The same result was found in R^S^Ф1- compared to R^R^. No significant differences (*p* > 0.05), instead, were detected between R^W^ and R^R^ groups or R^S^Ф1- and R^W^Ф1-, thus addressing the mentioned differences to phage metabolism. On the contrary, R^S^Ф1- and R^W^Ф1- groups were found to show significant differences (*p* < 0.05) in the concentration of the amino acid tyrosine, the alcohol ethanol and fatty acids, due to the different behavior of Ф1 when excised from the phage-resistant or sensitive strain.

These results suggest that lysogeny affects bacterial metabolism and confers to R^R^ biological advantages, which reflect both an increased fitness and the acquisition of virulence factors, implicated in modulating the host response (**Supplementary Figure S5**). Thus, taken together, genomics and metabolomics approaches allow for a complete understanding of the R^R^ physiology and its interplay with the host, providing essential information for the development of new candidate vaccines (61).

### Under-acylated LPS mitigates bacterial virulence in mice

**Figure 5:**
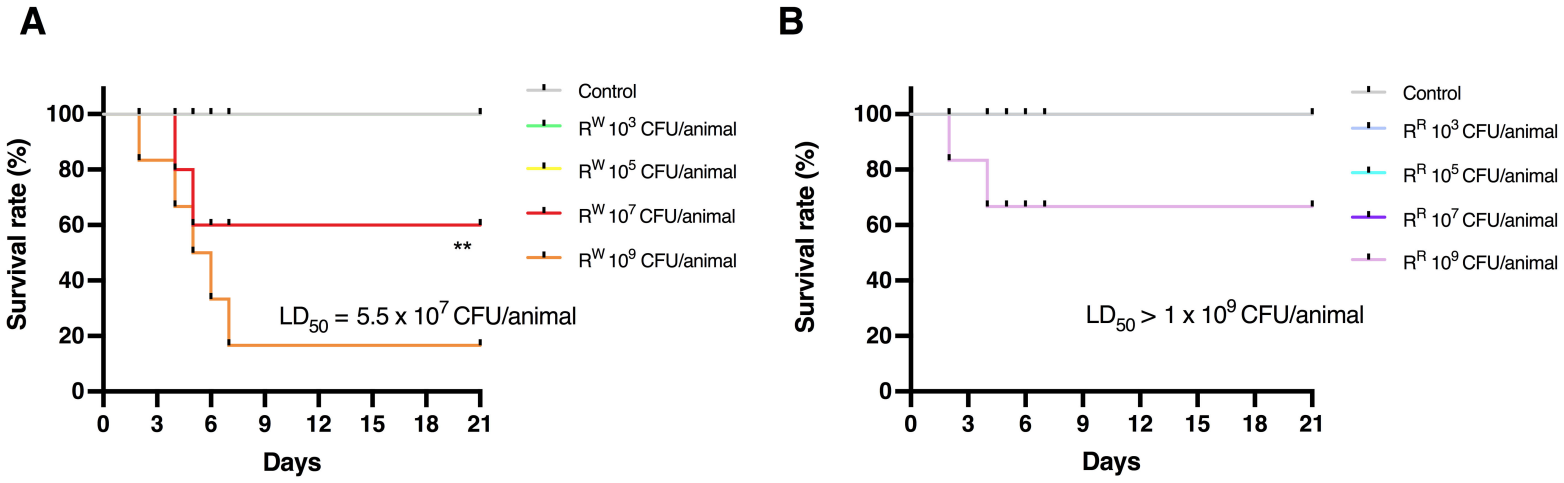
Determination of 50% Lethal Dose (LD_50_) of R^R^ and R^W^ in BALB/c mice. Survival curves of mice infected with different concentrations of R^W^ strain **(A)** or R^R^ strain **(B)**. Uninfected control group included mice injected with PBS vehicle (n = 6/group). ** *p* < 0.01 (Long-rank Mentel-Cox test). Data are representative of two independent experiments. LD_50_ was calculated according to the Reed and Muench method. LD_50_ = Log_10_ end-point bacterial dose = Log_10_ (bacterial dose showing mortality next below 50%) + {[(50% - mortality at bacterial dose next below 50%)/(mortality at bacterial dose next above 50% - mortality at bacterial dose next below 50%)] x Log_10_ (differences between bacterial doses used in the assay)]}.

To evaluate the effects of lysogeny on the virulence profile of *S.* Rissen, the lethal dose 50 (LD_50_) of both R^R^ and R^W^ strains was determined in a mouse model of infection. Female BALB/c mice aged 10 weeks were infected intraperitoneally with a dose-range of viable R^R^ or R^W^ bacteria (10^3^, 10^5^, 10^7^, 10^9^ CFU/mouse) and survival was monitored for 21 days (**Figure 5**).

**Figure 6:**
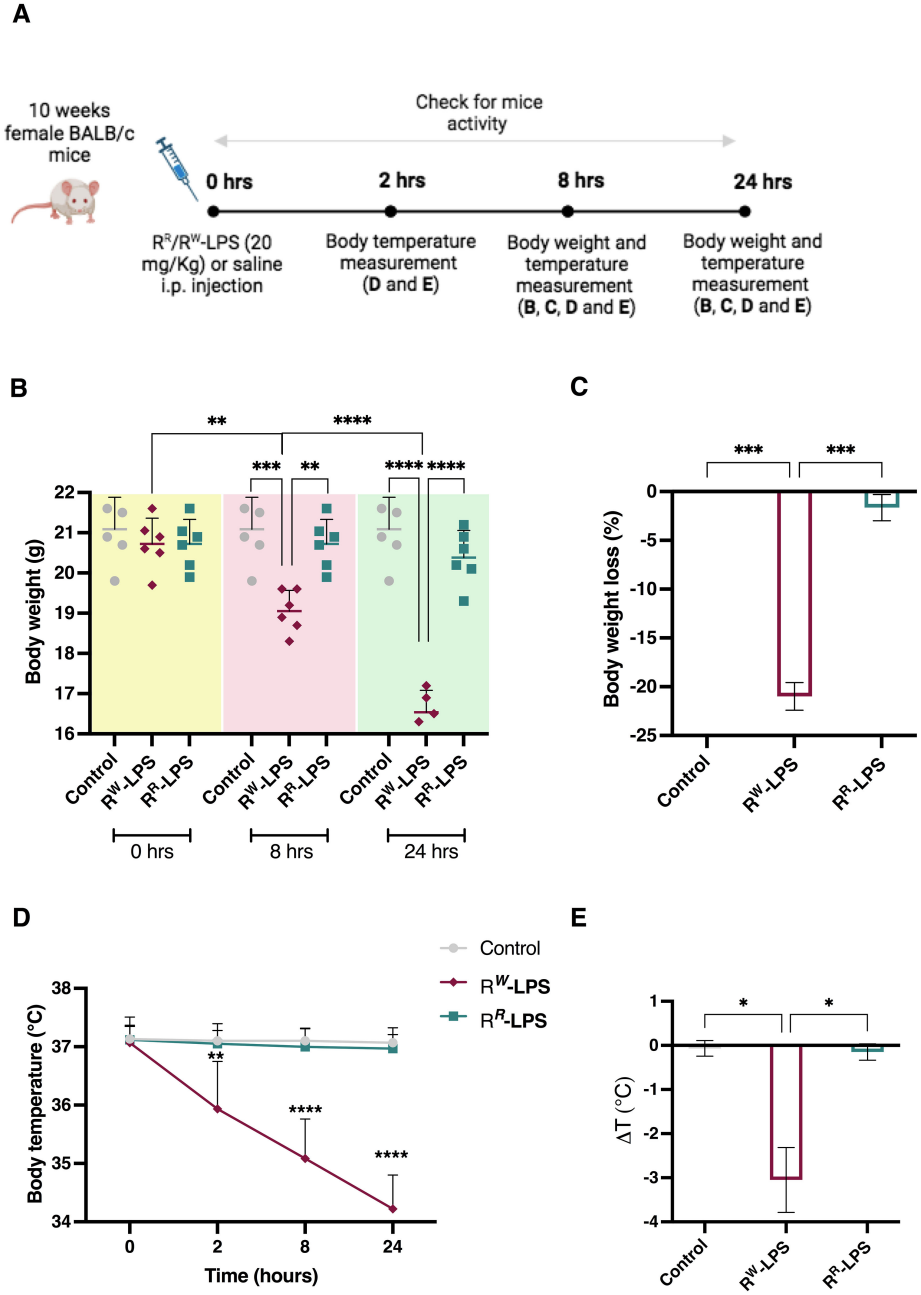
R^R^-LPS does not induce endotoxemia. **(A)** Experimental design for the intraperitoneal (i.p.) injection of R^R^ or R^W^-LPS. Ten-week-old, female BALB/c mice (n = 6 per group) were injected intraperitoneally with 20 mg/Kg of R^R^ or R^W^-derived LPS (R^R^-LPS and R^W^-LPS, respectively) dissolved in PBS solution and then body weight and temperature were evaluated. Mice (n = 6) in the control group (Control) were injected intraperitoneally with the same volume of PBS solution. **(B)** Body weight was measured 0, 8 and 24 hours after LPS stimulation. Graph represents mean ± SD of values from individual mice (dots). **(C)** Bar graph represents changes (%) of body weight 24 hours after LPS injection. Data are representative of a single experiment. Two-way ANOVA followed by Turkey *post-hoc* correction was used to compare body weight among three groups (Control, R^R^-LPS and R^W^-LPS) at different times. One-way ANOVA followed by Turkey *post-hoc* correction was used to compare body weight changes among three groups. **p < 0.01; ***p < 0.001; **** *p* < 0.0001. **(D)** Body temperature was measured 0, 2, 8 and 24 hours following LPS injection. **(E)** Bar graph represents change of temperature 24 hours after LPS injection. Data are representative of a single experiment. Two-way ANOVA followed by Turkey *post-hoc* correction was used to compare body temperature between R^R^-LPS and R^W^-LPS groups at different times following LPS injection. One-way ANOVA followed by Turkey *post-hoc* correction was used to compare the mean temperature among three groups. *p < 0.05; **p < 0.01; ****p < 0.0001.

Results displayed that mice infected with lower doses (10^3^ and 10^5^ CFU/animal) of *S.* Rissen – R^R^ or R^W^ – survived throughout the study (**Figure 5**; **Supplementary Table S3**). On the contrary, mice infected with higher doses (10^7^ and 10^9^ CFU/animal) showed different survival rates (**Figure 5**; **Supplementary Table S3**). Compared to R^R^-infected mice, a limited percentage (~30%) of mice infected with R^W^ (10^7^ CFU/animal) began dying 4 days after infection (**Figure 5B**). Furthermore, while 30% of mice infected with R^R^ (10^9^ CFU/animal) died 6 days after infection, almost all of the mice (~90%) were killed within 7 days following infection with the same amount of R^W^ (**Figure 5A**). These results show that the R^R^ strain displays a reduced virulence compared to R^W^, with a LD_50_ significantly higher (1 x 10^9^ CFU per mouse in R^R^ vs 5.25 x 10^7^ CFU per mouse in R^W^). This finding could find explanation in differences in the LPS-Lipid A structure between R^R^ and R^W^ (**Figure 2**). LPS is an endotoxin responsible for the host innate immune response activation, which can result in detrimental effects, such as the endotoxic shock (62). Due to its capacity to synthesize a LPS molecule lacking one or two acyl chains in the Lipid A portion, R^R^ only triggers a mitigated immune response with reduced risk for adverse secondary complications (63). This was confirmed by stimulating mice with a single intraperitoneal injection of LPS (20 mg/Kg body weight) purified from the R^R^ and R^W^ strains (R^R^-LPS and R^W^-LPS, respectively) and monitoring clinical signs of endotoxemia (**Figure 6A**). As expected, mice stimulated with R^W^-LPS displayed more severe signs of disease (decreased body temperature and weight, diminution of appetite and apathy) compared to the R^R^ one (**Figures 6B–E**), suggesting that mice are hyporesponsive to R^R^-LPS stimulation and hence, unable to mount an immediate defensive response. This property may reflect the unique organization of the R^R^-LPS and its capacity to mitigate the host immune system.

### Under-acylated LPS triggers a mitigated innate immune response in mice

**Figure 7:**
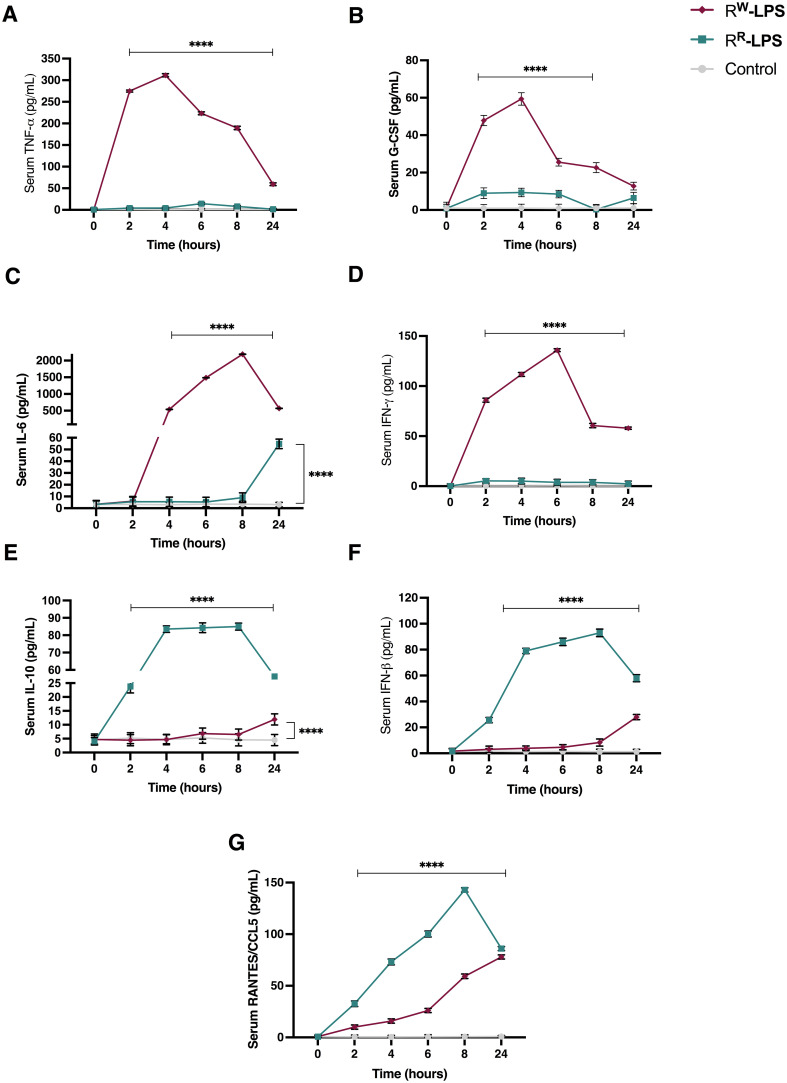
Cytokine levels in serum of BALB/c mice stimulated with R^R^ or R^W^-LPS. Mice (n = 6 per group) were injected intraperitoneally with 20 mg/Kg of R^R^ or R^W^-LPS dissolved in PBS solution. Mice in the control group (Control) were injected intraperiteonally with the same volume of PBS solution. Serum was collected every 2 hours for 8 hours and 24 hours following injection. The cytokines: **(A)** TNF-alpha; **(B)** G-CSF; **(C)** IL-6; **(D)** IFN-gamma; **(E)** IL-10; **(F)** IFN-beta and **(G)** RANTES/CCL5 were analyzed by ELISA. Data are representative of two independent experiments. Two-way ANOVA followed by Turkey *post-hoc* correction was used to compare cytokine levels among the three groups (control, R^R^- and R^W^-LPS groups). ****p < 0.0001.

To assess whether the biochemical diversity in R^R^ and R^W^-derived LPS affects the host innate immune response, we measured the expression levels of pro-inflammatory cytokine (TNF-α, G-CSF, IL-6, IL-10, IFN-β, RANTES/CCL5 and IFN-γ) using an ELISA assay. LPS (20 mg/Kg body weight) was injected intraperitoneally in female BALB/c mice (n = 6 per group) and blood samples were collected both every 2 hours for a period of 8 hours and at 24 hours post injection. Results showed a much stronger increase in TNF-α, G-CSF and IL-6 levels in blood serum of mice stimulated with R^W^-LPS compared to those stimulated with R^R^-LPS (**Figures 7A–C**).

TNF-α and G-CSF increased rapidly during the first hours of stimulation and reached their highest level 4 hours post-stimulation, then started declining (**Figures 7A, B**). However, their concentration remained higher than the control group or mice stimulated with R^R^-LPS (**Figures 7A, B**). INF-γ and IL-6, instead, were secreted later than TNF-α and G-CSF. The highest levels of these cytokines were observed 6- and 8-hours post-stimulation, respectively and, started decreasing after 24 hours of stimulation (**Figures 7C, D**). As expected, increased levels of IL-10 were detected at 24 hours, compared to control group (**Figure 7E**). These results show that R^W^-LPS initiates sooner an acute inflammatory response via the MyD88-dependent pathway (64), aimed at controlling the injury, as indicated by IL-10 up-regulation at 24 hours after LPS stimulation (**Figure 7E**). On the contrary, R^R^-LPS induced a milder inflammatory response. As illustrated in **Figure 7**, the secretion of the pro-inflammatory cytokines involved in the acute phase response (TNF-α and IL-6) was unaltered during the first hours post-LPS stimulation, compared to the control group (**Figures 7A, C**), while RANTES, IFN-β and IL-10 were found increased compared to both control group and mice injected with R^W^-LPS (**Figures 7E–G**). However, IL-6 showed an increase at 24 hours (**Figure 7C**). These data indicate the capability of the R^R^-LPS to selectively engage the TRIF-dependent pathway, while resulting a weaker elicitor of the MyD88-dependent pathway (65–67).

Taken together, these results suggest a different capacity of R^R^ and R^W^-LPS to stimulate the host immune response and a reduced toxicity of the R^R^-LPS.

### Under-acylated LPS stimulates a more efficient and safer antibody response

As a powerful antigen, LPS plays a pivotal role in immunological processes, stimulating antibody production (68). However, due to the Lipid A reactogenicity, LPS – in its native form - is not suitable as vaccine (69). Instead, the low toxicity of the R^R^-LPS (**Figure 7**) – resulting from the deacylation of Lipid A (**Figure 2**) – could make it safe enough to develop antimicrobial vaccines.

To investigate the protective role of the R^R^-LPS against *S.* Rissen infection, the immunization assay was performed. Therefore, we first explored the concentration of the whole LPS structure necessary for mice immunization, by evaluating the hemolytic effect (**Supplementary Figure S6**) of a wide range of doses (spanning from 1 to 500 μg). Results demonstrated that the minimum concentration of R^R^-LPS showing hemolytic effect < 8% was 10 μg. Thus, this concentration was selected to assess R^R^-LPS as a potential vaccine candidate.

**Figure 8:**
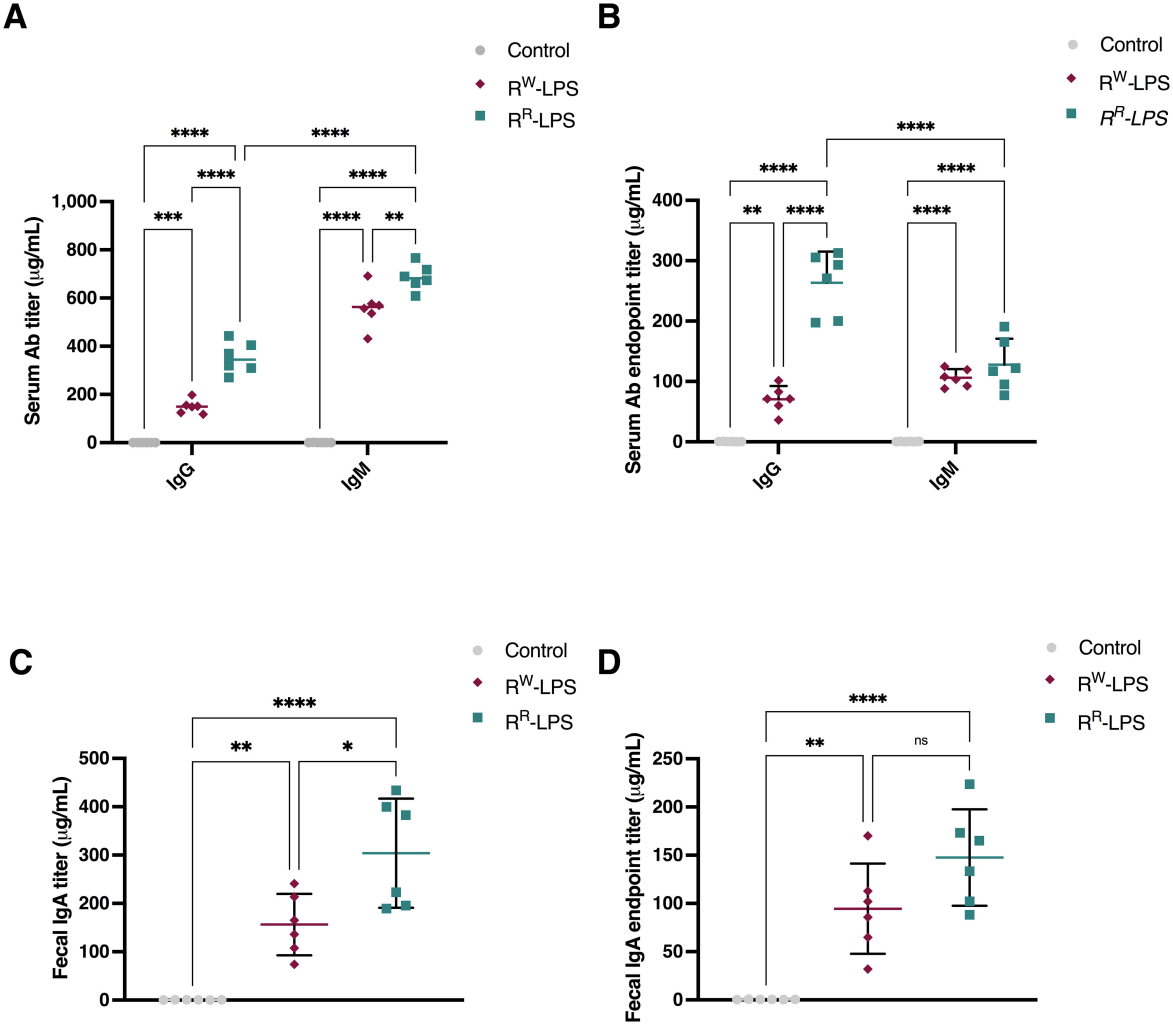
R^R^-LPS favors antibody response. **(A, C)** Total IgM, IgG and IgA antibody response elicited after immunization with R^R^-LPS or R^W^-LPS. Results are represented as mean titers (μg/mL) in serum or feces ± SD from female BALB/c mice (n = 6 per group) immunized intraperitoneally three times with: 1) R^R^-LPS (10 μg/100 μL PBS, teal green); 2) R^W^-LPS (10 μg/100 μL PBS, amaranth red) or 3) vehicle (100 μL PBS, grey). Graph represents mean ± SD of values from individual mice (dots). Data are representative of a single experiment. Two-way ANOVA followed by Turkey *post-hoc* correction was used to compare total antibody levels among three groups. * *p* <0.05; **p <0.01; ****p <0.0001. **(B, D)** IgM, IgG and IgA end-point anti-R^R^-LPS or R^W^-LPS antibody titers. Results represent the antibody response elicited on day 7 after last immunization and are represented as mean titers (μg/mL) in serum or feces ± SD. Data are representative of a single experiment. Two-way ANOVA followed by Turkey *post-hoc* correction was used to compare total antibody levels. ** *p* <0.01; ****p <0.0001. ns, not statistically significant.

To evaluate the level of the antibody response, two groups of BALB/c mice (6 mice per group) were immunized intraperitoneally with RSA-conjugated R^R^-LPS or R^W^-LPS (10 μg/100 μL PBS) twice, at an interval of two weeks, while a third group received an intraperitoneal (i.p.) dose of PBS (control group). The secondary booster was administrated seven days after the primary one. Mice immunized with R^R^-LPS exhibited higher levels of IgG and IgM (IgM > IgG) than those immunized with R^W^-LPS (**Figure 8A**). Of note, 7 days after the last immunization, the IgG response prevailed on the IgM one, with a higher extent in mice immunized with R^R^-LPS compared to those immunized with R^W^-LPS (**Figure 8B**). This result reflects the capability of R^R^-LPS to selectively engage the TLR4-TRIF-dependent pathway compared to R^W^-LPS (70). We also analyzed the IgA titers, as the primary antibody produced in the intestinal mucosa (71), site of *Salmonella* infection. Significant LPS-specific IgA titers were detected in feces of mice immunized with R^R^-LPS compared to both R^W^-LPS immunized mice and control ones (**Figure 8C**). Additionally, similar to IgM, IgA levels were found to decrease after the boosting doses (**Figure 8D**).

These results clearly suggest R^R^-LPS as a more effective and safer immunostimulant than the parent R^W^-LPS, as it retains immunostimulatory properties while lacking most of its toxicity.

### Under-acylated LPS protects mice against *Salmonella* Rissen infection

To evaluate the protective efficacy of the R^R^-LPS induced immunity against *S.* Rissen infection, all mice were infected with a bacteremia-inducing dose of the R^W^ strain (10^9^ CFU/animal) two weeks after the last immunization. The bacterial count in the splenic tissue was measured on day 4 post infection. Results revealed a significant reduction in bacterial burden in the spleens of mice immunized with R^R^-LPS, compared to both control mice and mice immunized with R^W^-LPS (**Table 1**).

**Table 1:** R^R^-LPS reduces bacterial burden in mice infected with *S.* Rissen.

	R^W^ (CFU/spleen)	
	UNCONJUGATED ANTIGEN	CONJUGATED ANTIGEN
R^R^-LPS	2 x 10^5^	< 10^3^
R^W^-LPS	5 x 10^7^	2.5 x 10^5^

Conversely, mice that received unconjugated R^R^-LPS exhibited only a modest reduction in bacterial burden. Although bacterial burden was still higher than in those receiving PBS or a single dose of R^W^-LPS, it was not lethal (**Table 1**).

**Figure 9:**
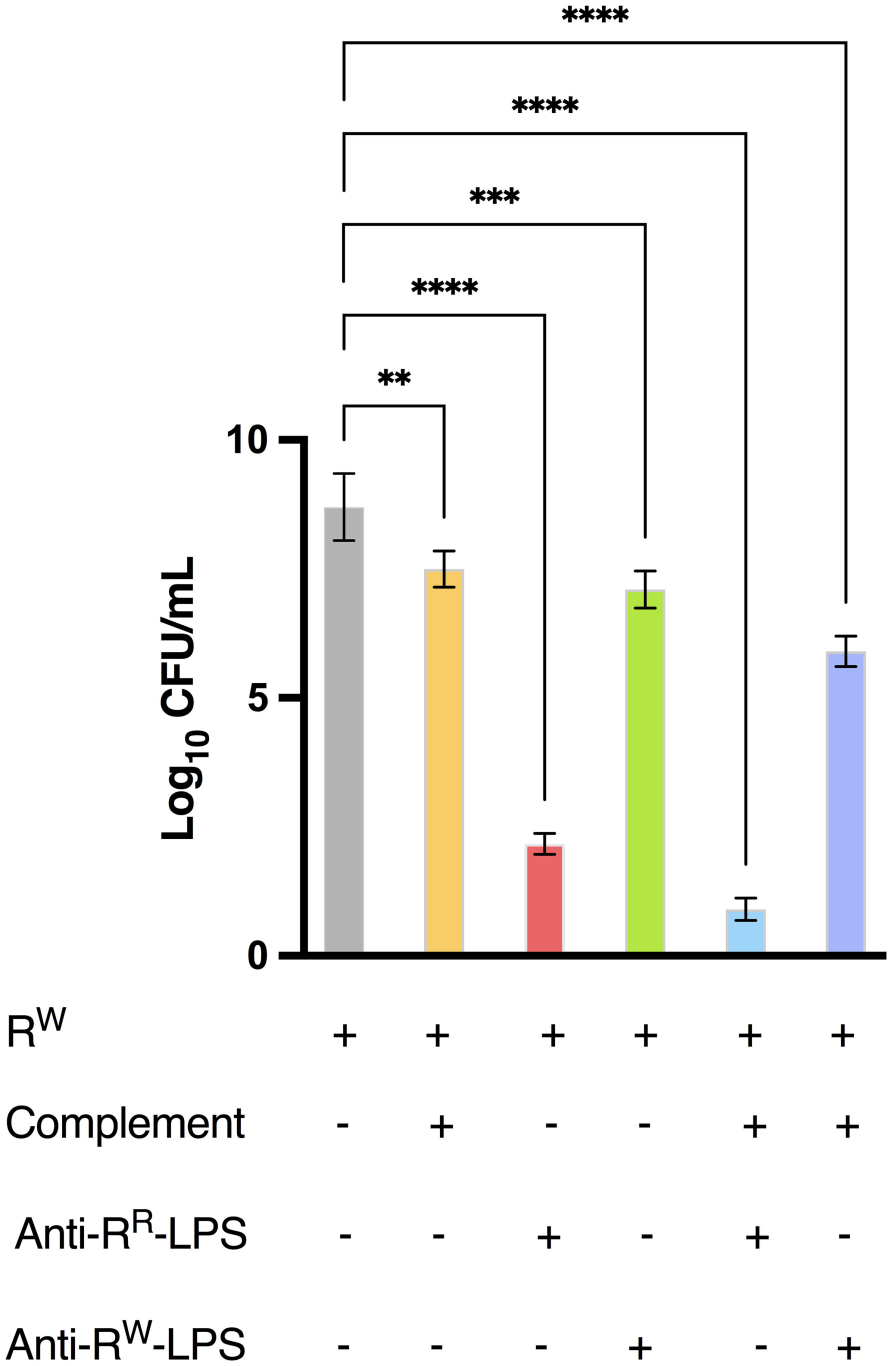
Antibodies from mice immunized with R^R^-LPS display bactericidal activity against WT *S.* Rissen. The bacteriophage-sensitive strain of *S.* Rissen (R^W^, 1 x 10^6^ CFU/mL) was incubated at 37°C for 3 hours alone, with purified antibodies derived from mice immunized with R^R^- or R^W^-LPS, with complement or with both purified antibodies and complement. Data are represented as Log_10_ of CFU/mL (OD_600_ = 1 = 5 x 10^8^ CFU/mL) and represent means ± SD of three independent experiments, each performed in triplicate. One-way ANOVA followed by Turkey *post-hoc* correction was used to compare Log_10_ CFU/mL among different experimental conditions. **p < 0.01; ***p < 0.001; **** *p* < 0.0001.

Collectively, these findings suggest that LPS-specific antibodies significantly contribute to immune protection against *S.* Rissen infection, as confirmed by serum bactericidal activity (**Figure 9**) and highlight R^R^-LPS as a promising adjuvant for the development of vaccines against *Salmonella* infections.

### Under-acylated LPS-specific antibodies mediate cross-reactivity

*Salmonella* species share a high degree of homology in genes encoding cell-surface molecules, including LPS, a major target of antibodies (20). As a result, immunization with LPS from one *Salmonella* serovar could confer immunity against related serovars.

To explore the potential of anti-R^R^-LPS antibodies to confer cross-protective immunity to closely related *Salmonella* serovars, mice were intraperitoneally immunized at two-week intervals with 100 μL of R^R^-LPS (10 μg) or vehicle (sterile PBS). Four days after the final immunization, blood samples were collected, and serum was separated by centrifugation. Serum samples were then used to test cross-reactivity between anti-R^R^-LPS antibodies and LPSs from different *Salmonella* serovars through an agglutination test. As shown in **Supplementary Table 4**, serum samples (undiluted or diluted 1:50 and 1:100) exhibited variable agglutination with different selected *Salmonella* strains (*S.* Infantis*, S.* Newport and *S.* Choleresius). Specifically, undiluted serum showed strong agglutination with S. Choleraesuis and *S.* Infantis, which are both part of the *S.* Rissen serogroup C1. When diluted 1:50 or 1:100, serum also agglutinated the above *Salmonella* serovars, although the reaction required more time than the undiluted serum. Remarkably, a late agglutination activity of undiluted serum was observed against *S.* Newport, expressing O:6,8 antigens (C2 serogroup).

The strong interaction of anti-R^R^-LPS antibodies with S. Choleraesuis and *S.* Infantis and, to a lesser extent with *S.* Newport, suggests the O-antigenic determinants O:6 and 7 as their major target. These results indicate that anti-R^R^-LPS antibodies may confer cross-protection against related *Salmonella* serovars by targeting shared O-antigens.

### Under-acylated LPS-specific antibodies mediate host protection by targeting the O-antigen

The O-antigen portion of LPS is widely recognized as the key target for immune defense against bacterial infections.

To further determine whether O-antigen is the primary antigenic determinant recognized by anti-R^R^-LPS antibodies, an indirect ELISA assay was performed. Serum samples from mice immunized with R^R^-LPS were tested for reactivity against both the O-antigen and LPS from the R^R^ and R^W^ strains. Results revealed that anti-R^R^-LPS antibodies interacted with both LPS and O-antigen from the two *S.* Rissen strains (**Supplementary Table 5**). Specifically, the antibodies showed moderately stronger reactivity with LPS and O-antigen from the R^R^ strain than with those from the R^W^ strain. Notably, LPS exhibited slightly stronger reactivity than the purified O-antigen (*p* < 0.05, **Supplementary Table 5**).

These results demonstrate that O-antigen is the primary R^R^-LPS epitope responsible for modulating the antibody-mediated host protection, supporting our previous findings (**Supplementary Table 4**). Furthermore, they suggest that other, poorly investigated portions of the LPS molecule may also contribute to the host defense and antibody reactivity.

### Discussion

In this study, we investigated the protective effect of an LPS-based vaccine against *S.* Rissen in BALB/c mice. LPS is a key immunogenic component of Gram-negative bacteria. It stimulates the host humoral immune response and provides protection against pathogens (72, 73). However, LPS also plays an essential role in bacterial virulence through its endotoxic component (Lipid A), which raises concerns about its safety as “subunit” vaccine in its native form.

Several efforts have been made to attenuate the Lipid A toxicity while preserving the LPS immunostimulatory properties. Mutations in the *msbB* and *PagP* genes, both involved in the Lipid A acylation pattern, have been shown to reduce the capacity of bacteria to induce a pro-inflammatory response in the host (22, 74). As a result, *msbB* and *PagP* mutant strains are considered promising candidates for vaccine development, particularly in the context of outer membrane vesicle (OMV)-based vaccines (75). However, despite this, *Salmonella msbB* mutants display growth defects and increased sensitivity to environmental stressors such as physiological CO_2_ levels and low pH (76, 77), limiting their survival and complicating large-scale vaccine production.

In this study, we propose - for the first time - phage resistance as a promising biological tool for producing under-acylated variants of LPS, overcoming the limitations mentioned above.

Phage-resistance, as a naturally occurring selection mechanism, bypasses the need for microbial genetic manipulation, thus avoiding challenging related to the stability of mutations and the maintenance of the desired phenotypic traits. Moreover, by enhancing bacterial fitness, phage-resistance promotes bacterial survival under stress conditions, offering a cost-effective and sustainable alternative to conventional genetic, chemical and enzymatic methods.

To explore the role of prophages in remodeling Lipid A molecules and modulating *S.* Rissen virulence, we first examined the presence of phage genetic elements in the R^R^ and R^W^ genomes. Both strains contained phage genome elements. However, R^R^ acquired the superinfection exclusion gene (*SieB*), which confers a competitive advantage increasing bacterial fitness by inhibiting further phage infections (78) (**Supplementary Figure S1**; **Figure 1**). This superinfection resistance likely arises from alterations in cell membrane elements, such as the LPS and its endotoxic portion (30). Modifications to the LPS structure may occur at each step of its biosynthetic process, as well as from additional enzymes (79). We recently showed that R^R^ expresses higher levels of the *LpxR* gene than R^W^ (26). *LpxR* encodes a deacylase responsible for removing acyl chains from Lipid A. Therefore, we hypothesized the capability of R^R^ of producing deacylated Lipid A species.

In the present study, we validated the above hypothesis. Mass spectrometry analysis revealed that, unlike the R^W^ strain, R^R^ produces a higher abundance of two alternative variants of Lipid A as the penta-acylated and the tetra-acylated form (**Figure 2**). Both these two structures are less stimulatory for TLR4 and elicit a mitigated innate immune response. These results demonstrate that the acquisition of the *SieB* gene, which is associated with the distinctive phage-resistant phenotype of R^R^, improves bacterial fitness and provides virulence factors (LpxR) which attenuate the innate immune response in the eukaryotic host.

Metabolic changes in bacteria often influence the production of virulence factors and contribute to bacterial pathogenicity (80). Short chain fatty acids (SCFAs; propionate, acetate and butyrate) regulate the expression of genes in *Salmonella* pathogenicity island 1 (*SPI1*, 81). Specifically, acetate promotes *SPI1* genes expression, while butyrate suppresses genes like *hilD*, which plays transcriptional regulatory roles (81). Notably, *hilD* also activates *LpxR* (82). In line with these findings, R^R^ uptook from the growth medium more acetate than R^W^ (**Supplementary Figure S5**), suggesting that phage-resistance enhances the expression of virulence factors like LpxR, which regulate LPS structure and modulate the host innate immune response. This result is confirmed by our previously published data which demonstrate up-regulation of the *LpxR* gene in AGS cells infected by the R^R^ strain (26). Additionally, R^R^ implemented the extracellular concentration of γ-aminoisobutyric acid (GABA), glucose and pyruvate compared to R^W^ (**Supplementary Figure S5**). GABA improves bacterial survival in acidic environments and supports colonization of gastro-intestinal tract (83). Similarly, accumulation of glucose in the growth medium, together with pyruvate production from acetate, confer advantages to bacteria, preserving them from acidic environments (84). These findings suggest that phage Ф1 provides R^R^ with an adaptive advantage by enhancing bacterial fitness.

As living organisms, bacteria ordinarily require energy to support their physiological functions. However, under particular conditions - such as the acquisition of phage-resistance - bacteria demand higher levels of energy. In agreement with this requirement, R^R^ uptook more hypoxanthine from the growth medium than R^W^ (**Supplementary Figure S5**). As an intermediate of the purine biosynthetic pathway, hypoxanthine consumption increases the ATP production to provide energy for the more metabolically active R^R^ cells. R^R^ also uptook more amino acids, including arginine, lysine, isoleucine, leucine, proline and phenylalanine which serve as effective source of carbon and nitrogen (**Supplementary Figure S5**, 85). Amino acids contribute to ammonium production, which is vital for synthetizing macromolecules through glutamate biosynthesis (86). Consistent with this evidence, R^R^ released glutamate into the growth medium (**Supplementary Figure S5**), which also participates in glutathione biosynthesis – a key antioxidant molecule that counteracts reactive oxygen species (ROS) produced by the host immune system (87). Higher levels of methionine - the major precursor of glutathione (88) - were detected in the growth medium of R^R^ (**Supplementary Figure S5**), aligning with our previous results showing increased expression of the *SodC1* and *gogB* genes in cells infected by the R^R^ strain (26). *SodC1* is a superoxide dismutase which protects bacteria from the oxidative burst induced by the host immune cells, while *gogB* attenuates both the inflammatory response and ROS production (28). Taken together, these results suggest that phage-resistance, at least in this case, enhances bacterial fitness and reduces bacterial effectiveness in inducing cell host damage.

These results were confirmed *in vivo*. R^R^, in fact, curbs the mortality of mice. The highest tested concentration of R^R^ (10^9^ CFU/animal) resulted in 33% mortality in mice, compared to 100% mortality in those exposed to the same concentration of R^W^ (**Figure 5**). This reduced mortality may reflect under-acylation of Lipid A and consequently reduced activation of the innate immune response.

Under-acylated Lipid A molecules, in fact, compete with native species of Lipid A (hexa-acylated forms) and inhibit the hexa-acylated Lipid A-MD2 complex functions, thus inducing a deficient TLR4 signaling and a reduced secretion of pro-inflammatory cytokines (58).

As expected, mice stimulated with R^R^-LPS produced lower levels of pro-inflammatory cytokines, compared to those stimulated with the R^W^-LPS (**Figure 7**). They showed lower concentrations of the MyD88-dependent cytokines TNF-α, IL-6 and G-CSF (**Figures 7A–C**) – all involved in the early stages of infection – thus suggesting the inability of R^R^ in mounting an immediate inflammatory response. Importantly, R^R^-LPS suppressed the IFN-γ production (**Figure 7D**), which plays a key role in the pathogenesis of septic shock (89), while increased the production of TRIF-dependent cytokines RANTES and IFN-β (**Figures 7F–G**), which limit inflammation and promote adaptive immunity (65, 66). Alteration in the stability of TLR4-MD-2, due to under-acylated ligands, in fact, impairs the canonical LPS-induced receptor dimerization and, as a consequence, the traditional signal transduction pathway involving both MyD88 and TRIF-pathways. These results, together with the secretion of the anti-inflammatory cytokine IL-10 (**Figure 7E**), explain reduced signs of endotoxemia in mice stimulated with the R^R^-LPS (**Figure 6**).

To determine the protective effect of the R^R^-LPS against *S.* Rissen *in vivo*, we immunized mice and evaluated the bacterial count in the splenic tissue. A significant reduction in bacterial burden (< 10^3^) was observed in mice immunized with the R^R^-LPS compared to those immunized with the R^W^-LPS (**Table 1**). This protection was attributed to LPS-specific antibodies targeting the O-antigen (**Supplementary Table S5**; **Figure 9**). Both R^R^-LPS and R^W^-LPS induced an early production of IgA, with a significant higher expression in mice immunized with R^R^-LPS (**Figure 8C**).

IgA is the most important class of antibodies released in the intestinal secretion and plays an essential role in protecting the gastro-intestinal tract from *Salmonella* pathogens (90). Moreover, early IgA production – as in our case – represents a valuable immunological memory to polysaccharidic antigens (91, 92),. In addition, mice immunized with the R^R^-LPS also showed an immune response profile characterized by higher production of IgG than those immunized with the R^W^-LPS (**Figures 8A, B**). As a selective activator of the TRIF-dependent pathway, R^R^-LPS induces the recruitment of the adaptive immune response through the expression of *type I-IFN*-encoding genes and IL-10 production (**Figure 7**), which stimulate both maturation and differentiation of CD80^+^ and CD86^+^ dendritic cells through selection and expansion of pathogen-specific T and B cells (93). These events culminate in the T-dependent antibody production – enhanced by the peptide carrier, which enables the antigen presentation to T cell through the MHCII – thus eliciting a protective immunological memory through the antibody switching (93, 94), and explaining the higher expression of IgG in R^R^-LPS immunized mice. Altogether, these results give reason for attributing the host immune protection to the antibody production (**Figure 9**) and, hypothesizing that R^R^-LPS immunization could confer cross-protection against different *Salmonella* serovars by targeting shared O-antigens (**Supplementary Table 4**). However, further in-depth investigation is needed to elucidate the exact mechanism of protection and ascertain whether antibodies are the unique contributors to the R^R^-LPS induced immune protection.

In conclusion, this study demonstrates that under-acylated LPS molecules - derived from the bacteriophage-resistant strain of *S.* Rissen - induce an attenuated innate immune response, while promoting an effective adaptive immune protection. These properties make R^R^-LPS a potentially promising candidate for vaccine development. Moreover, this study also highlights the potential use of phages as an alternative to conventional chemical or genetic methods for producing vaccines. Nevertheless, further studies are required to provide further insight into mechanism, length and contribution of additional factors in improving vaccine potency and efficacy, and finally, the possibility to extend this approach to other bacterial infections.

## Data Availability

The datasets presented in this study can be found in online repositories. The names of the repository/repositories and accession number(s) can be found in the article/**Supplementary Material**.
