# Supplementary material for: Phage-resistance alters Lipid A reactogenicity: a new strategy for LPS-based conjugate vaccines against Salmonella Rissen
Source: Front Immunol. 2024 Dec 11;15:1450600. doi: 10.3389/fimmu.2024.1450600 (PMC11668645; doi:10.3389/fimmu.2024.1450600)
Supplement: Supplementary file 1 [file DataSheet1.docx]

Supplementary Material

Phage-resistance alters Lipid A reactogenicity: a new strategy for LPS-based conjugate vaccines against *Salmonella* Rissen

**Paola Cuomo^1,4,†,^ Chiara Medaglia^2,†^, Angela Casillo^3^, Antonio Gentile^1,4^, Carmine Fruggiero^1^, Maria Michela Corsaro^3^, Rosanna Capparelli^1,4*^**

*** Correspondence:** Rosanna Capparelli: capparel@unina.it

# Supplementary Figures and Tables

This file includes: Supplementary Figures (1-6) and Supplementary Tables (1-5).

**Supplementary Figures**

**
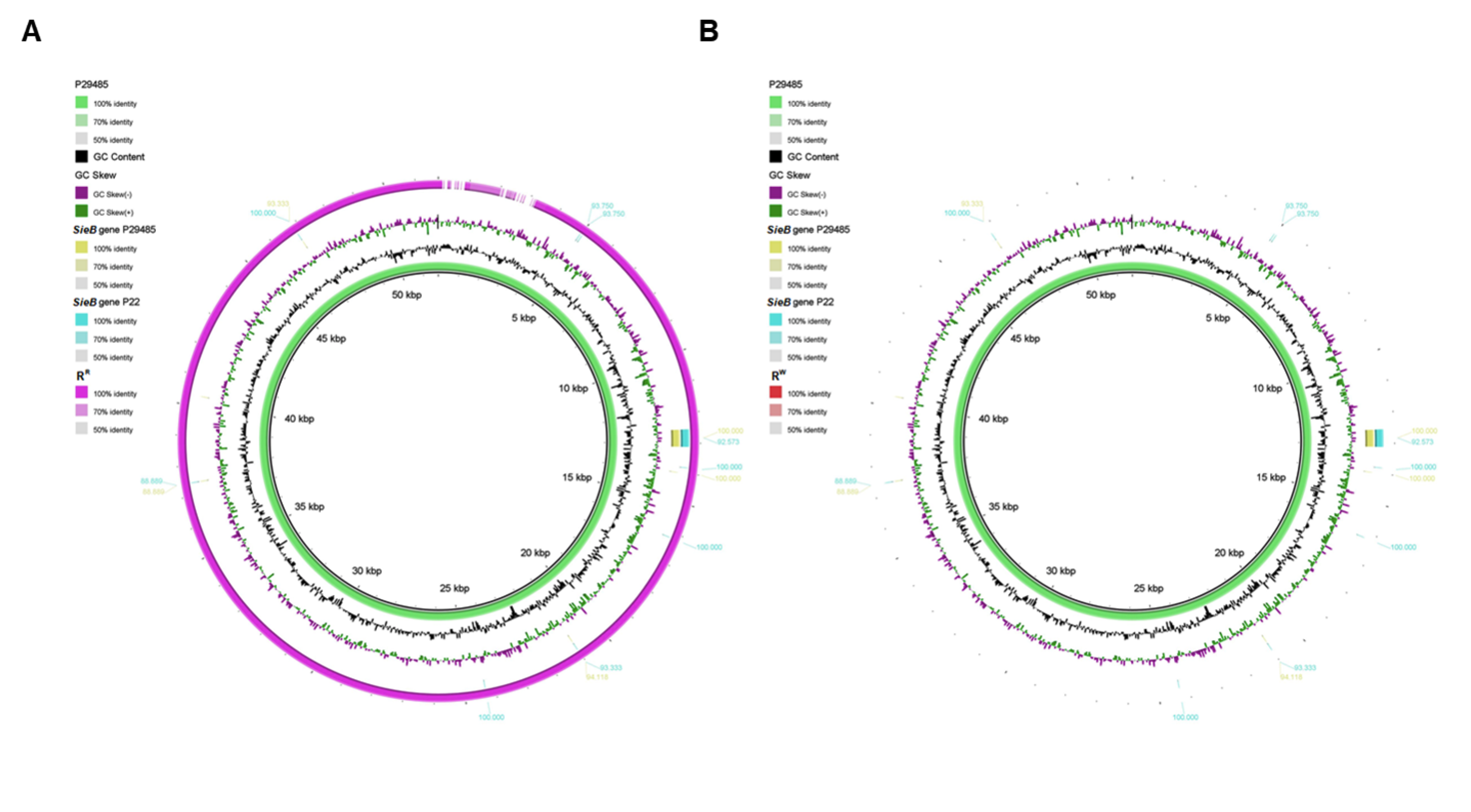
Supplementary Figure 1.** Genomic comparison between R^R^ and R^W^. Both R^R^ genome (**A**) and R^W^ genome (**B**) were aligned to the prophage P29485 (Φ1) genome, in order to detect the presence of the *SieB* gene, which was assigned using P22 prophage genome as reference.

**A**

***Salmonella* Rissen phage 29485**

MNNSWWQELMHFFLQGMTLKQLIHMLIILILLIVVMPVSVKEWVNLHNPEILPQYWMYYILLFCVSYVLN

GVVNSVYHAVNERIEASTAQQRKAREEKVVRDLFDSLTPGERAYLAFAVAANNQLKTEKGSPESISLLEK

GLITRLPSVIGYPDIDRFVIPEKYFNECYMRFAGKSDILMNELIAQDEQLKK

***Salmonella Typhimurium* phage P22**

MNNSWWQELMRFFLQGMTLKQLIHMLIILIVLIIVMPVSVKEWINLHNPEILPHYWMYYILLFCVSYVLN

GVVNSVYHAVTERIEASTAQRRKDREEKVVRDLFDSLTLGERAYLAFAVAANNQLKTEKGSPEAISLLKK

GIITRLPSAIGYPDIDRFIIPEKYFNECYMRFAGKSDILMNELIVQDEQLKK

**B**

Query 1 MNNSWWQELMHFFLQGMTLKQLIHMLIILILLIVVMPVSVKEWVNLHNPEILPQYWMYYI 60

MNNSWWQELM FFLQGMTLKQLIHMLIILI+LI+VMPVSVKEW+NLHNPEILP YWMYYI

Sbjct 1 MNNSWWQELMRFFLQGMTLKQLIHMLIILIVLIIVMPVSVKEWINLHNPEILPHYWMYYI 60

Query 61 LLFCVSYVLNGVVNSVYHAVNERIEASTAQQRKAREEKVVRDLFDSLTPGERAYLAFAVA 120

LLFCVSYVLNGVVNSVYHAV ERIEASTAQ+RK REEKVVRDLFDSLT GERAYLAFAVA

Sbjct 61 LLFCVSYVLNGVVNSVYHAVTERIEASTAQRRKDREEKVVRDLFDSLTLGERAYLAFAVA 120

Query 121 ANNQLKTEKGSPESISLLEKGLITRLPSVIGYPDIDRFVIPEKYFNECYMRFAGKSDILM 180

ANNQLKTEKGSPE+ISLL+KG+ITRLPS IGYPDIDRF+IPEKYFNECYMRFAGKSDILM

Sbjct 121 ANNQLKTEKGSPEAISLLKKGIITRLPSAIGYPDIDRFIIPEKYFNECYMRFAGKSDILM 180

Query 181 NELIAQDEQLKK 192

NELI QDEQLKK

Sbjct 181 NELIVQDEQLKK 192

**Supplementary Figure 2.** Protein sequence comparison between SieB protein of *S.* Rissen phage 29485 (Φ) and *S.* Typhimurium phage P22. (**A**) SieB proteic sequence of both *S.* Rissen phage 29485 (Φ) and *S.* Typhimurium phage P22. (**B**) Sequence alignment of the UniProt entries ARB10858 and AAF75022 by the Basic Local Alignment Search Toll (BLAST). Query 1 represents *S.* Rissen phage 29485, while Subject 1 represents *S.* Typhimurium phage P22. In yellow are highlighted amino acids which differ between the two sequences.


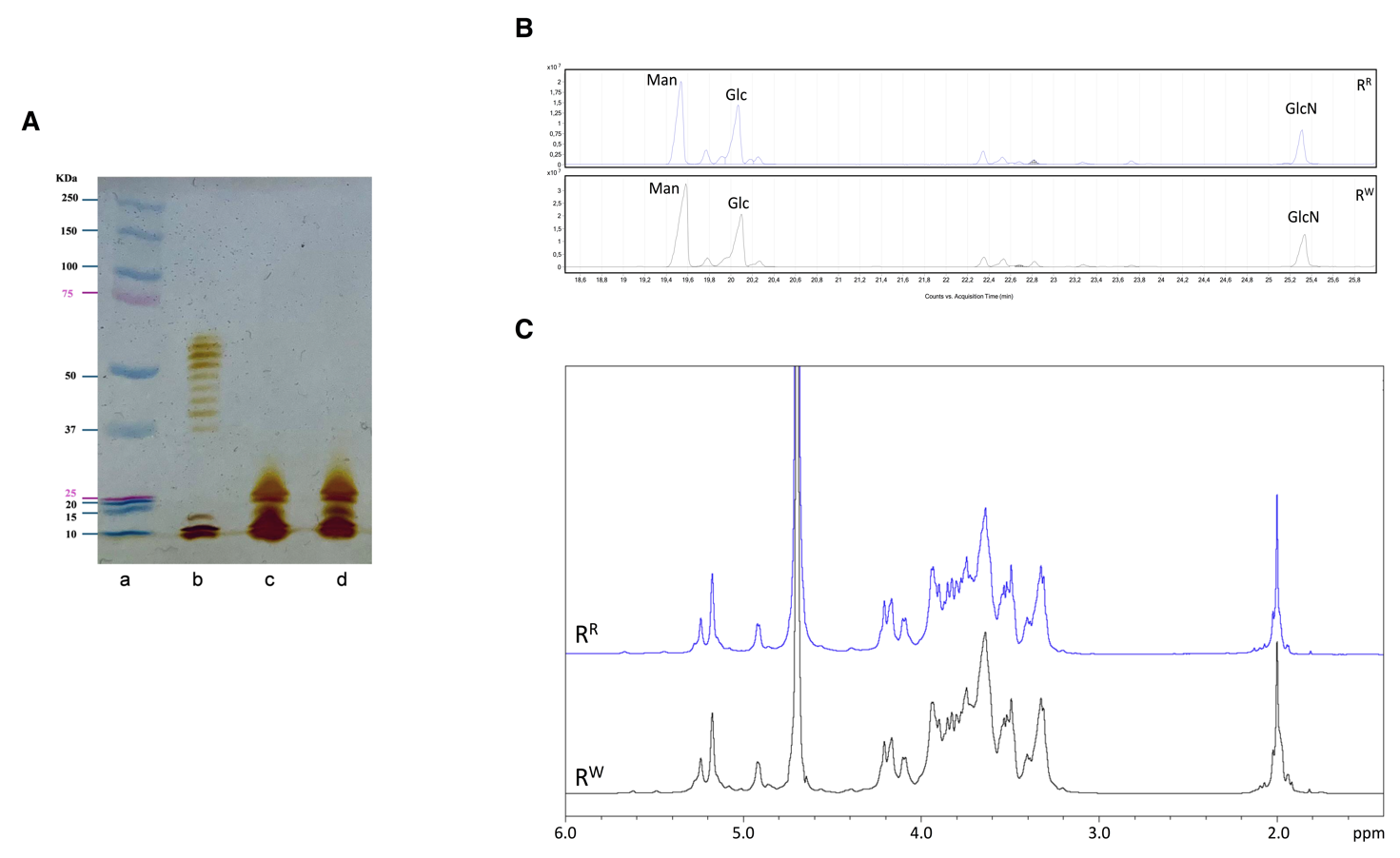
**Supplementary Figure 3.** R^R^- and R^W^-LPS chemical differences. (**A**) 14% DOC-PAGE analysis, stained with silver nitrate, of LPS extracted from R^R^ and R^W^ *S.* Rissen strains. (**Lane a**) Precision Plus Protein Dual Color Standards (4µL); (**Lane** **b**) LPS from *E. coli* O111:B4 (8µL) has been used as a standard; (**Lane** **c**) purified LPS from *S.* Rissen R^R^ (8µL); (**Lane** **d**) purified LPS from *S.* Rissen R^W^ (8µL). (**B**) Gas-chromatography Mass-Spectrometry (GC-MS) chromatogram of AMGs of the OPSs from R^R^ and R^W^ strains. Mannose (Man), Glucose (Glc), and Glucosamine (GlcN). (**C**) Overlapped ^1^H NMR spectra of the OPS isolated from *S.* Rissen R^R^ and R^W^. Spectra were acquired at 298K, in D_2_O at 600 MHz.

**
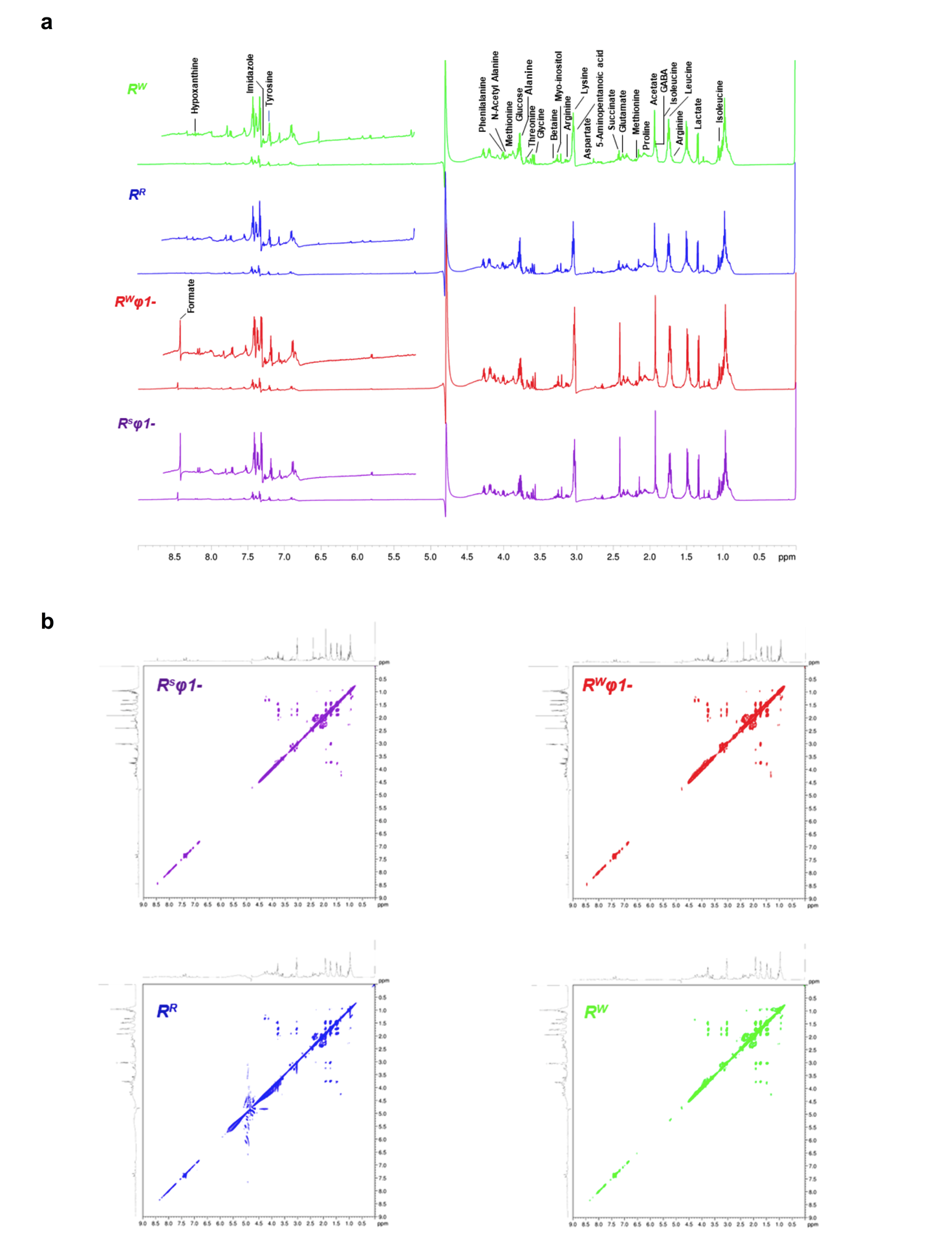
Supplementary Figure 4.** ^1^H **(A)** and ^1^H-^1^H **(B)** NMR spectra of the growth medium from the phage-sensitive and -resistant strain of *S.* Rissen before and after phage excision (R^W^; R^R^; R^W^Φ1– and R^S^Φ1, respectively).

**A**

**B**


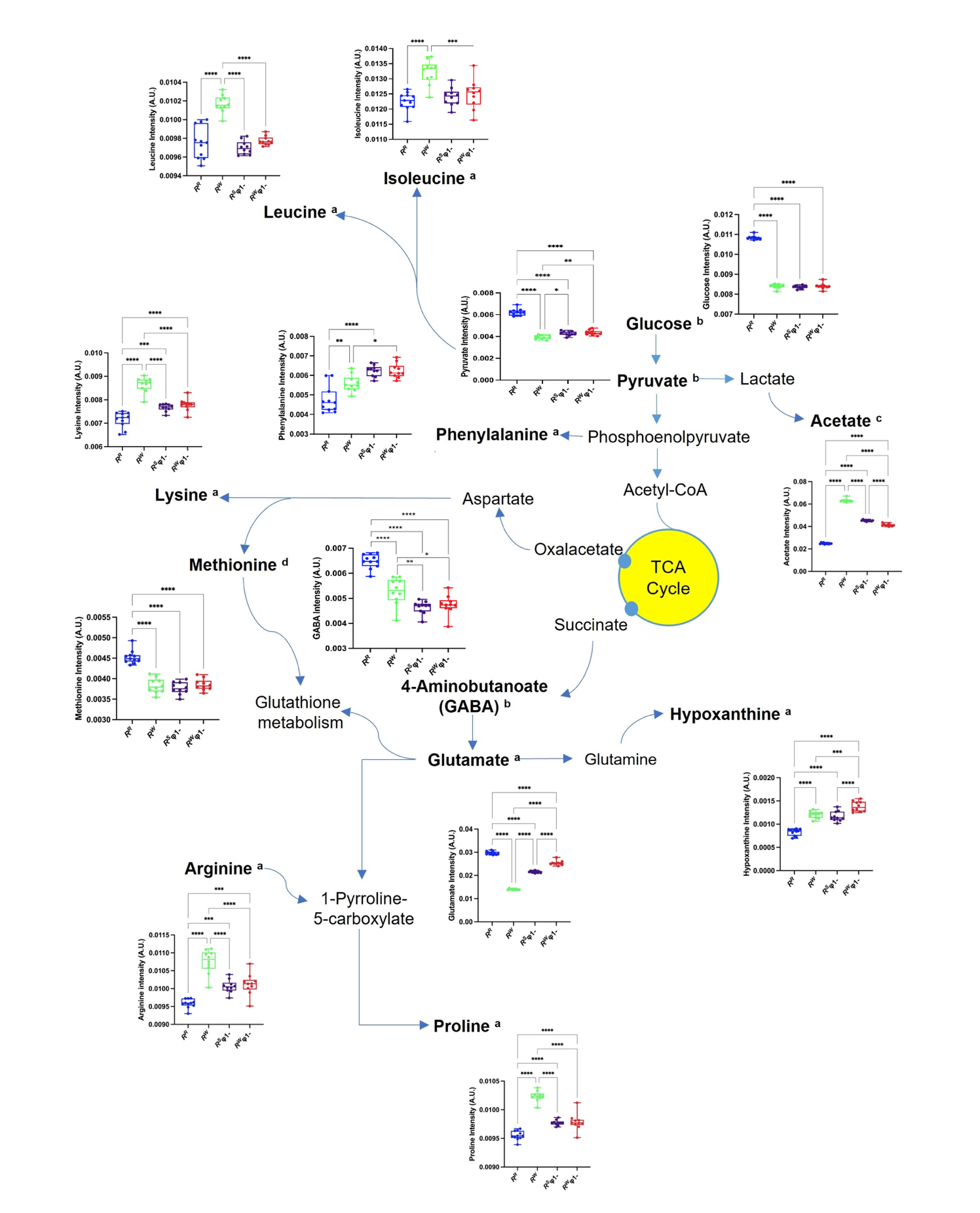


**Supplementary Figure 5.** Schematic overview of extracellular metabolite changes associated with phage-resistance. Metabolite intensity is represented as whisker plot and is reported as mean ± SD of values from individual replicates (dots). Data are representative of an individual experiment performed in replicates (ten replicates for each conditions). Two-way ANOVA, followed by Bonferroni post-hoc correction test, was used to perform multiple comparisons. *, *p* < 0.05; **, *p* < 0.01; ***, *p* < 0.001; ****, *p* < 0.0001. **a** Metabolites used in energy production; **b, d** metabolites used in increasing bacterial fitness; **c** metabolite used in increasing virulence by promoting LPS modification.

**
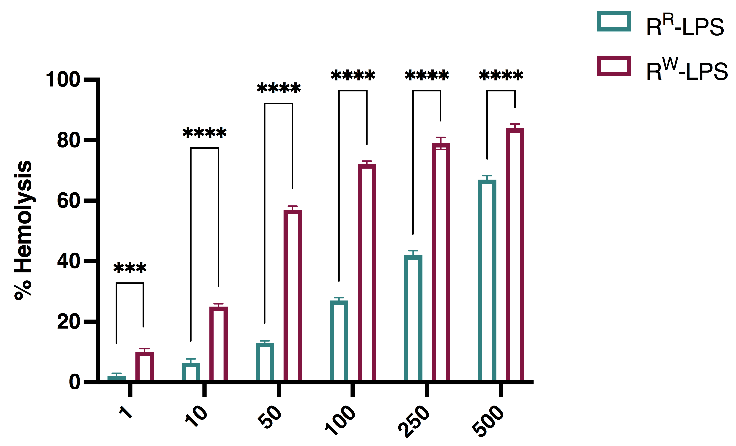
**

**Supplementary Figure 6.** Hemolytic effect of R^W^ and R^R^-LPS on human red blood cells. Graph bars represent mean ± SD of two individual experiments performed in triplicate. Results are reported as a percentage of hemolytic effect compared to untreated cells. A wide range of R^R^ or R^W^-LPS concentrations was tested (from 1 to 500 μg/mL). Unpaired and two-tailed t-test was used to compare two treatments at each data set. ***, *p* < 0.001; ****, *p* < 0.0001.

**Supplementary Tables**

**Supplementary Table 1.** Partially methylated alditol acetate of OPSs from R^R^ and R^W^ strains.

| Derivative | Deduced Linkage |
| --- | --- |
| 1,5-di-*O*-acetyl-2,3,4,6-tetra-methyl hexitol | *t*-Glc*p* |
| 1,2,5-tri-*O*-acetyl-3,4,6- tri-methyl hexitol | 2-Man*p* |
| 1,2,3,5-tetra-*O*-acetyl-4,6-di-methyl hexitol | 2,3- Man*p* |
| 1,3,5-tri-*O-*acetyl-(2-deoxy-2-*N*-methyl acetamide)-  -4,6di-methyl hexitol | 3-GlcN*p* |

**Supplementary Table 2.** ^1^H and ^13^C chemical shift assignments of metabolites of the R^R^, R^W^, R^S^Φ1- and R^W^Φ1- growth medium.

| **METABOLITE** | **GROUP** | **δ ^1^H (ppm)** | **δ ^13^C (ppm)** | **^1^H MOLTEPLICITY** | **NMR PEAK INTENSITY (A.U.)** | | | |
| --- | --- | --- | --- | --- | --- | --- | --- | --- |
|  |  |  |  |  | **R^R^** | **R^W^** | **R^SΦ1-^** | **R^WΦ1-^** |
| Acetate | *β*CH_3_ | 1.92 | 24.07 | singlet | 0.0247  ±0.0006 | 0.0632  ±0.0014 | 0.0452  ±0.0006 | 0.0413  ±0.001 |
| Alanine | *α*CH | 3.80 | 51.10 | quadruplet | 0.00666  ±0.0001 | 0.00538  ±6.5E-05 | 0.00542  ±4.8E-05 | 0.00544  ±4.6E-05 |
| 4-Aminobutanoate | *β*CH_2_ | 1.88 | 24.54 | quintet | 0.00648  ±0.0003 | 0.00528  ±0.0005 | 0.004641  ±0.0002 | 0.00474  ±0.0004 |
| 5-Aminopentanoic acid | *δ*CH_2_ | 3.02 | 34.78 | triplet | 0.00806  ±0.0001 | 0.00966  ±0.0001 | 0.00893  ±9.9E-05 | 0.00904  ±9.5E-05 |
| Arginine | *γ*CH_2_ | 1.68 | 24.40 | multiplet | 0.00959  ±0.0001 | 0.0107  ±0.0003 | 0.0101  ±0.0002 | 0.0101  ±0.0003 |
| Aspartate | *β'*CH | 2.80 | - | doublet-doublet | 0.00248  ±7.9E-05 | 0.00116  ±0.0002 | 0.00127  ±0.0001 | 0.00126  ±0.0001 |
| Betaine | *δ*CH_2_ | 3.27 | 66.7 | multiplet | 0.00238  ±0.0003 | 0.00202  ±0.0003 | 0.00282  ±0.0002 | 0.00283  ±0.0003 |
| Formate | HCOO^–^ | 8.46 | - | singlet | 0.0008  ±0.0001 | 0.00153  ±0.0004 | 0.00202  ±0.0002 | 0.00181  ±0.0002 |
| Glycine | *α*CH | 3.58 | 42.00 | singlet | 0.00457  ±7.2E-05 | 0.00359  ±0.0001 | 0.00363  ±8.5E-05 | 0.00376  ±0.0001 |
| Glucose | C6H | 3.76 | 61.70 | doublet | 0.0108±  9.9E-05 | 0.00840±  0.0001 | 0.00837±  7.4E-05 | 0.00840±  0.0001 |
| Glutamate | *γ*CH_2_ | 2.34 | 34.00 | triplet | 0.0296  ±0.0007 | 0.0139  ±0.0002 | 0.0216  ±0,0004 | 0.0253  ±0.001 |
| Hypoxanthine | NH-C=O | 8.22 | - | singlet | 0.000828±  7.2E-05 | 0.00120±  7.6E-05 | 0.00117±  0.0001 | 0.00138±  0.0001 |
| Isoleucine | *γ*^’^CH | 1.46 | - | multiplet | 0.0123  ±0.0003 | 0.0132  ±0.0004 | 0.0124  ±0.0003 | 0.0125  ±0.0005 |
| Imidazole | C1H | 7.26 | 129.27 | singlet | 0.000802  ±0.0003 | 0.00102  ±0.0001 | 0.00110  ±0.0001 | 0.000899  ±0.00017 |
| Lactate | *β*CH_3_ | 1.32 | 20.76 | doublet | 0.00513  ±0.0001 | 0.00580  ±0.0001 | 0.00609  ±5.1E-05 | 0.00606  ±0.0001 |
| Leucine | *β*CH_2_ | 1.72 | 40.50 | multiplet | 0.00973  ±0,0001 | 0.0102  ±9.9E-05 | 0.00971  ±7.1E-05 | 0.00978  ±4.8E-05 |
| Lysine | *ε*CH_2_ | 3.01 | 39.50 | triplet | 0.00715  ±0.0003 | 0.00865  ±0.00035 | 0.00767  ±0.0001 | 0.00779  ±0.0003 |
| Methionine | *β*CH_2_ | 2.18 | 30.50 | - | 0.00452  ±0.0002 | 0.00383  ±0.0002 | 0.00377  ±0.0001 | 0.00385  ±0.0001 |
| myo-Inositol | C5H | 3.29 | 75.13 | triplet | 0.00164  ±0.0001 | 0.00163  ±0.0002 | 0.00210  ±0.0001 | 0.00194  ±0.0002 |
| N-acetyl Alanine | *β*CH_3_ | 1.36 | - | doublet | 0.7  ±0.0003 | 0.00514  ±0.0002 | 0.00628  ±0.0001 | 0.00632  ±0.0002 |
| Nicotinate | C5H | 8.62 | 149.95 | doublet-doublet | 0.000272  ±0.0001 | 0.000415  ±0.0002 | 0.000897  ±0.0001 | 0.000753  ±0.0002 |
| Phenylalanine | *α*CH | 4.00 | 56.80 | doublet-doublet | 0.00475  ±0.0007 | 0.00559  ±0.0004 | 0.00623  ±0.0003 | 0.00623  ±0.0004 |
| Pyruvate hydrate | *β*CH_3_ | 1.50 | - | singlet | 0.00622  ±0.0003 | 0.00393  ±0.0002 | 0.00427  ±0.0002 | 0.00436  ±0.0002 |
| Proline | *γ*CH_2_ | 2.02 | 23.90 | multiplet | 0.00955±  7.9E-05 | 0.0102±  9.3E-05 | 0.00977±  4.8E-05 | 0.00978±  0.0001 |
| Succinate | *β*CH_2_ | 2.42 | 34.96 | singlet | 0.00588  ±0.0009 | 0.0190  ±0.0008 | 00229  ±00004 | 0.0219  ±0.0009 |
| Threonine | *α*CH | 3.60 | 61.00 | doublet | 0.00525  ±0.0003 | 0.00503  ±0.0002 | 0.00459  ±0.0001 | 0.00458  ±0.0002 |
| Tyrosine | C2,6 aromatic | 7.18 | 130.00 | doublet | 0.000495  ±0.0002 | 0.000959  ±0.0001 | 0,000718  ±0.0001 | 0,000996  ±0.0001 |

**Supplementary Table 3.** Determination of 50% Lethal Dose (LD_50_) of bacteriophage-resistant (R^R^) and sensitive (R^W^) *S.* Rissen in BALB/c mice.

| **Bacterial strain** | **Infectious dose (CFU/animal)** | **Death/Total** | **OBSERVED**  **VALUES**  **Died Survived** | | **CUMULATIVE VALUES**  **Died Survived Total** | | | **MORTALITY**  **Ratio Percentage** | |
| --- | --- | --- | --- | --- | --- | --- | --- | --- | --- |
| R^W^ | 1 x 10^3^ | 0/6 | 0 | 6 | 0 | 17 | 17 | 0/17 | 0% |
|  | 1 x 10^5^ | 0/6 | 0 | 6 | 0 | 11 | 11 | 0/11 | 0% |
|  | 1 x 10^7^ | 2/6 | 2 | 4 | 2 | 5 | 7 | 2/7 | 28.6% |
|  | 1 x 10^9^ | 5/6 | 5 | 1 | 7 | 1 | 8 | 7/8 | 87.5% |
| R^R^ | 1 x 10^3^ | 0/6 | 0 | 6 | 0 | 22 | 22 | 0/22 | 0% |
|  | 1 x 10^5^ | 0/6 | 0 | 6 | 0 | 16 | 16 | 0/15 | 0% |
|  | 1 x 10^7^ | 0/6 | 0 | 6 | 0 | 10 | 10 | 0/10 | 0% |
|  | 1 x 10^9^ | 2/6 | 2 | 4 | 2 | 4 | 6 | 2/6 | 33.3% |

**Supplementary Table 4.** Agglutination test of the R^R^ pAb solution with different *Salmonella* serovars.

| **ANTIGEN** | ***Salmonella* Rissen Ab solution** | | |
| --- | --- | --- | --- |
|  | Undiluted | 1:50 | 1:100 |
| *Salmonella* Rissen | +++ | +++ | +++ |
| *Salmonella* Choleraesuis | +++ | ++ | ++ |
| *Salmonella* Infantis | +++ | ++ | ++ |
| *Salmonella* Newport | +/- | +/- | - |

+++ = Agglutination within 1 minutes.

++ = Agglutination within 3 minutes.

+ = Agglutination within 5 minutes.

+/- = Reduced agglutination within 5 minutes.

**Supplementary Table 5.** Serum Ab reactivity from mice immunized with the R^R^-LPS against O-antigen and LPS of both R^R^ ad R^W^ strains.

| ***Salmonella* Rissen** | **Indirect ELISA (OD_450_ nm)** | |
| --- | --- | --- |
|  | O-antigen | LPS |
| R^R^ | 2.82 ± 0.052 | 2.98 ± 0.011 |
| R^W^ | 2.44 ± 0.019 | 2.53 ± 0.033 |

Results are expressed as OD (450 nm) values ± SD of a single biological experiment performed in triplicate.
